# Discrete network models of endothelial cells and their interactions with the substrate

**DOI:** 10.1007/s10237-023-01815-1

**Published:** 2024-02-14

**Authors:** Raphael Jakob, Ben R. Britt, Costanza Giampietro, Edoardo Mazza, Alexander E. Ehret

**Affiliations:** 1 Institute for Mechanical Systems, ETH Zurich, CH-8092 Zürich, Switzerland; 2 Empa, Swiss Federal Laboratories for Materials Science and Technology, CH-8600 Dübendorf, Switzerland

**Keywords:** Random networks, Cytoskeleton, Endothelial monolayers, Traction force microscopy, LAMMPS, Finite element model

## Abstract

Endothelial cell monolayers line the inner surfaces of blood and lymphatic vessels. They are continuously exposed to different mechanical loads, which may trigger mechanobiological signals and hence play a role in both physiological and pathological processes. Computer-based mechanical models of cells contribute to a better understanding of the relation between cell-scale loads and cues and the mechanical state of the hosting tissue. However, the confluency of the endothelial monolayer complicates these approaches since the intercellular cross-talk needs to be accounted for in addition to the cytoskeletal mechanics of the individual cells themselves. As a consequence, the computational approach must be able to efficiently model a large number of cells and their interaction. Here, we simulate cytoskeletal mechanics by means of molecular dynamics software, generally suitable to deal with large, locally interacting systems. Methods were developed to generate models of single cells and large monolayers with hundreds of cells. The single-cell model was considered for a comparison with experimental data. To this end, we simulated cell interactions with a continuous, deformable substrate, and computationally replicated multistep traction force microscopy experiments on endothelial cells. The results indicate that cell discrete network models are able to capture relevant features of the mechanical behaviour and are thus well-suited to investigate the mechanics of the large cytoskeletal network of individual cells and cell monolayers.

**Supplementary Information:**

The online version contains supplementary material available at 10.1007/s10237-023-01815-1.

## Introduction

The endothelial monolayer (EML) consists of a single layer of endothelial cells (EC). It forms the innermost layer of blood and lymph vessels (Vajda et al 2021), and thus, it is the only biological tissue which is in direct contact with blood or the lymphatic fluid under physiological conditions. As a part of the cardiovascular system, ECs are exposed to various biological, chemical, and mechanical cues. Alteration of these cues may be associated with a large group of cardiovascular diseases, which are still a globally leading cause of death (Roth et al 2018). In many of these diseases, EML injury is part of, or even the major event in the pathological process. For example, tearing of the EML is an important step for the subsequent deposition of plaque in atherosclerotic arterial walls (Lee 2020). Vice versa, EML integrity can also be impaired during the treatment of cardiovascular diseases, such as the deployment of vascular stents or artificial heart valves, which can damage the EML with subsequent complications (Autar et al 2020). In addition to the pivotal role in cardiovascular diseases, the EML is also essentially involved in the event of cancer metastatisation as it forms the interface through which tumour cells exit the vascular system (Amos and Choi 2021). The mechanisms by which cancer cells weaken the intercellular connection between ECs in a monolayer are an active topic of research, with potentially important implications for effective cancer treatment (Wang et al 2022; Hamester et al 2022).

While biological and chemical cues represent key factors for EML homeostasis and pathology, the governing event for rupture of the monolayer is a critical mechanical load. Such critical loads can either result from a decrease in mechanical resilience under otherwise physiological loads or, vice versa, from an increase in mechanical loads to pathological levels. For this reason, the mechanical characterisation of the EML under physiological, supraphysiological, and pathological conditions is highly relevant for further advancing the understanding of factors affecting EML integrity, associated medical implications, and potential therapeutic approaches.

Different from the majority of other body cells, ECs form a dense monolayer without extracellular matrix in between the cells. Consequently, the EML mechanical characteristics are governed by the intracellular cytoskeleton, its connections with other cells, and the interactions with the cell substrate. Information about the cytoskeletal structure and properties can be gained by appropriate experimental setups. Advanced microscopy techniques, micro-needle experiments, and traction force microscopy (TFM) are among frequently used instruments for characterisation of cytoskeletal properties (Øie et al 2018; Matthews et al 2004; Lekka et al 2021). Nevertheless, quantitative information about the mechanical properties of the single cytoskeletal members of the cell is scarce, although particularly the understanding of intracellular force distributions seems highly relevant for the study of complex, mechanically induced signalling cascades. The complexity of these mechanotransduction events calls for a characterisation of the propagation of forces from the cellular membrane and substrate connections through the cytoskeleton to the nucleus.

Given the current limitations in experimental techniques, computational models of both cells and EMLs have been established to support the experimental work. Particularly in the field of cytoskeletal dynamics powerful software tools have been developed in the recent past, such as Cytosim (Nedelec and Foethke 2007; Belmonte et al 2017; Pensalfini et al 2023), MEDYAN (Popov et al 2016), AFINES (Freedman et al 2017), and aLENS (Yan et al 2022) among others (see, e.g. Yan et al 2022, for an overview), able to account for the interaction of the cytoskeletal members and cross-linkers. To simulate cells at larger scales and their mechanical interaction with the environments, other approaches have been taken, which entail reduced computational cost. A widely used technique relies on representing the single cells in a continuous fashion within a finite element (FE) framework with only some of the intracellular components considered as discrete members. These models are usually three-dimensional and include representations of the nucleus, cytoplasm, membrane, nuclear membrane, and cytoskeletal components (e.g. Barreto et al 2013; Bansod et al 2018; Khunsaraki et al 2021; Banerjee et al 2021; Shen et al 2020; Jakka and Bursa 2021; Stracuzzi et al 2021). Typically, the cytoplasm and nucleus are represented as volume elements, whereas the membrane and nuclear membrane are represented by membrane or shell and the cytoskeleton by beam or truss elements. Such models often incorporate the principles of tensegrity (Ingber 2003), proposing presence of tensile and compressive members within the cytoskeleton that are in equilibrium with each other.

More detailed models of the cytoskeleton were considered, e.g. including actin cortex, actin filaments, intermediate filaments (IF), and microtubules (MT) to simulate cell nanoindentation and cellular adhesion processes (Fang and Lai 2016), or incorporating actin fibres, actin-binding proteins, and membranous components to study the deformation of the nucleus during micropipette pulling (Zeng et al 2012). While the cytoskeletal resolution in three dimensions provided by these models might indeed be sufficient to address various mechanotransduction aspects, the resulting complexity also entails high computational cost. This prevents a manageable extension of the model from a single cell to multicellular monolayers.

A more efficient way of simulating cellular monolayers is provided by vertex models (e.g. Barton et al 2017; Noll et al 2017; Latorre et al 2018; Nestor-Bergmann et al 2018; Mosaffa et al 2018; Das et al 2021; Jensen and Revell 2023), in which each cell is usually portrayed as a convex polygon. The monolayer is formed by connecting the cell to its neighbours at the polygon’s border. Vertex models were successfully applied to address different aspects, e.g. the superelastic behaviour of cells under equibiaxial tension (Latorre et al 2018), or effects of active cortical tension (Noll et al 2017), and to simulate dynamic events such as cell growth, division, and apoptosis even in monolayers with tens of thousands of cells (Barton et al 2017). However, modelling different inter- and intra-cellular events is challenging. Recently, some models have bridged this gap by introducing a representative cytoskeletal component as well as intercellular junctions (Escribano et al 2019; McEvoy et al 2022). These models have focussed on the formation of intercellular gaps as opposed to intracellular events.

To date, there are only few monolayer models that explicitly consider intracellular components. A three-dimensional unit cell model including one-, two-, and three-dimensional elements patterned onto a substrate was established to represent a cell monolayer for studying endothelial transduction (Dabagh et al 2017). Xu et al (2022) built a two-dimensional coarse-grained monolayer model of epithelial cells, including intercellular interactions and generalised intracellular forces to analyse wound formation.

In general, computational costs limit either the level of intracellular complexity or the number of cells within a monolayer that can be considered. Therefore, numerical efficiency is a key factor in establishing models of monolayers both large enough to study corporate effects of the cells and detailed enough to consider the role of the intracellular components of individual cells. In the present work, we propose to address this problem by exploiting the computational capacity of molecular dynamics (MD) software. MD tools, originally designed to compute problems at atomistic length scales, have frequently been used beyond their original field of application, e.g. to represent lipid bilayers (Yuan et al 2010), or to investigate the mechanical properties of polymer networks via two-dimensional discrete network analysis (Alamé and Brassart 2019). Moreover, ‘peridynamics’ (Silling 2000) has been established as an alternative approach to continuum mechanics, and its application to fracture of lipid bilayers (Taylor et al 2016) suggests that it may serve to model cell components as peridynamic continua.

The specific network-like structure of the cytoskeleton suggests that its characteristics and interactions can be translated into the framework of atoms and bonds interacting through distance-dependent energetic potentials. In particular, if the cytoskeleton is considered a central force network (Picu 2011), the bonds can be interpreted as fibre links between the cross-links represented as atoms (cf. Britt and Ehret 2022). We show here that powerful models of individual cells can be generated by this approach. Individual cell components and interactions are represented as bond potentials in the framework of the Large-scale Atomic/Molecular Massively Parallel Simulator LAMMPS. In the same fashion, models of groups of cells are established towards a better understanding of mechanical signal propagation from the cell substrate through the membrane to the nucleus and finally within the monolayer. The development of the modelling framework is the focus of the present work. Existing data from multistep traction force microscopy are used for a first model validation.

## Methods

A summary of frequently used abbreviations is provided in Table 1.

### A brief description of the EC cytoskeleton

**Table 1:** List of abbreviations

2D	Two dimensional
BC	Boundary condition
BVP	Boundary value problem
CoV	Coefficient of variation
DNM	Discrete network model
$\varepsilon _{\textrm{A}}$	Substrate area strain
$\Delta \varepsilon _{\textrm{A}}$	Difference of area strain underneath and
	around the cell
EC	Endothelial cell
EML	Endothelial monolayer
FA	Focal adhesion
FE	Finite element
HUVEC	Human umbilical vein endothelial cell
IF	Intermediate filament
LAMMPS	Large-scale atomic/molecular massively
	parallel simulator
MD	Molecular dynamics
MT	Microtubule
SF	Stress fibre
STD	Standard deviation
TFM	Traction force microscopy

**Fig. 1:**
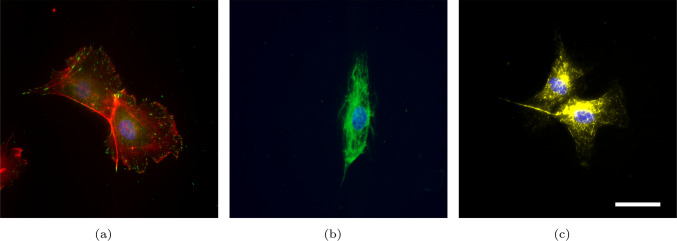
Confocal laser scanning microscopy images of the different components of the endothelial cytoskeleton: (a) adherent cell pair with actin (red), focal adhesions (phospho-paxillin, green), and nuclei (DAPI, blue) showing the intercellular connections (adherens junctions) between the actin networks. (b) Vimentin intermediate filament (green) and nucleus (DAPI, blue) of a single cell. (c) Alpha-tubulin network (yellow) and nuclei (DAPI, blue) of a cell pair. The scale bar in (c) refers to 25 $\mu$m and applies to all images

Figure 1 shows confocal laser scanning microscopy images of sparse human umbilical vein endothelial cells (HUVEC) stained to visualise the nucleus (blue), the focal adhesions (green in Fig. 1a), and the members of the cytoskeleton: filamentous actin (a), intermediate filaments (b), and microtubules (c).

Depending on the density and availability of crosslinkers, *filamentous actin* (f-actin) either assembles into branched network structures or into oriented filament bundles, forming long fibres (Lieleg et al 2010). Cortical actin expressed in network form lines the inner side of the cellular membrane (Chugh and Paluch 2018), while stress fibres (SF) span the EC body. They can carry high tensile loads and are able to contract actively. It is distinguished between ventral and dorsal SFs, transverse arcs, and the perinuclear cap (Burridge and Guilluy 2016) based on their location within the cell and their characteristics. Ventral SFs are located at the cellular floor near the substrate surface. Dorsal SFs similarly originate at the cellular floor but are then oriented towards the cellular roof, and are usually found at the leading edge of migrating cells. Transversal arcs bind to dorsal SFs, thereby generating a coarse network of SFs. Finally, the perinuclear cap consists of SFs that originate and end at the cellular floor, while enveloping the nucleus. The actin filaments of ventral SFs, the transverse arc, and the perinuclear cap are crosslinked with non-muscle myosin II, which induces contraction and hence ‘activation’ of the stress fibres in homeostasis. In contrast, dorsal SFs are mainly crosslinked by $\alpha$-actinin and not activated in homeostasis (Kovac et al 2013). The cortical actin as well as the SFs are connected to the substrate at protein complexes called focal adhesions (FA). Moreover, they are connected to their neighbouring cells in the monolayer by tight and adherens junctions (Hartsock and Nelson 2008).

The *intermediate filaments* consist of different proteins, the most abundant of which are vimentin and keratin (Liu et al 2010). The IFs form a dense network that binds to the nucleus and spreads throughout the cell to protein complexes called desmosomes and hemidesmosomes located in the cellular membrane (Broussard et al 2020). The desmosomes form intercellular connections and are located at the cell–cell interface, while hemidesmosomes bind to the substrate and are located at the cell–substrate interface. Their curvy appearance suggests that IFs are slack in the homeostatic cell (cf. Fig. 1(b)). Upon extension, they undergo conformational changes leading to nonlinear mechanical response exhibiting a force plateau and subsequent stiffening at high strains (Kreplak and Fudge 2007; Lorenz et al 2019). Hence, IFs are considered to be responsible for cellular toughness and increased stiffness under high loads whereas contributing little at low and moderate strains (Hu et al 2019; van Bodegraven and Etienne-Manneville 2021).

The *microtubules*, unlike SFs and IFs, do not bind to the nucleus but originate at the centrosome and span throughout the cell. They consist of tubulin and feature a hollow tube structure with significant cross-sectional area. The resulting flexural rigidity suggests that MTs can resist both tensile and compressive mechanical loads. Because stress fibres can bind to the MTs (Dogterom and Koenderink 2019), they were considered the compressive members in the tensegrity theory, while the stress fibres are the tensile members (Ingber 2003). Because of their low abundance compared to the other cytoskeletal members, they are not considered to increase the cell’s tensional rigidity significantly.

In the present work, we focus on the planar response of the cells and, therefore, omit the representation of dorsal SFs in the actin network. Moreover, since we consider the response of the cells under moderate tensile strains, both the IF and MT networks are neglected.

### Discrete cell models

Two-dimensional (2D) discrete random fibre network models (DNM) were generated with Python 3.7 (including the modules NumPy (v. 1.24.3), SciPy (v. 1.10.1), and Shapely (v. 2.0.1)) containing the boundary of the cellular membrane, and the projections of the boundary of the nuclear membrane, cortical actin, focal adhesions, ventral and perinuclear SFs, and nuclear actin onto the cellular floor.

#### Fibre network model of ECs

The cellular domain was prescribed by a convex polygon, specified through 6 points within a box of 100 $\mu$m $\times$ 100 $\mu$m. The polygon edges represent the boundary of the cellular membrane, which was split into several smaller linear segments. The nucleus was defined as an ellipse centred around a point within the polygon and characterised by its major and minor axes and their orientation. Its edge defines the boundary of the nuclear membrane, which once again was split into several smaller segments joined at nuclear membrane connection points. The nucleus contains nuclear actin filaments, which were represented by 50 random connections between two nuclear membrane points.

The actin cortex was created by first randomly seeding points in the cellular domain according to a Poisson disc algorithm (Bridson 2007), serving as seed points for SciPy’s Voronoi mesh generation. The network was connected to the cellular membrane at the membrane element connection points.

FAs were defined by a random selection of *n* nodes from the cortical actin mesh. Their number $n_{\textrm{FA}}$ ranging from 200 to 300 was selected consistent to values reported in the literature (Nair et al 2021). This resulted in a FA density of 0.04 $\textrm{FAs}/\mu \textrm{m}^2$, which is close to the values reported by Chala et al (2021).

A total of 35-40 ventral SFs were generated by connecting two FAs, randomly selected among pairs of FAs with distance $\ge 30$ $\mu$m, thus ensuring that the fibres span a substantial distance within a typical cell. Similarly, to generate perinuclear SFs, we chose connections between FAs that cross the vicinity of the nucleus but not the nuclear domain itself. A random subset of 10-20 of these connections was then selected and relayed to the nuclear membrane connection point in closest proximity to the direct line between the two FAs.

Clearly, the number of ventral and perinuclear SFs represents a free parameter in the model. The present choice is based on preliminary studies and the comparison of the corresponding model predictions with experimental results (Sec. 2.6.1).

**Fig. 2:**
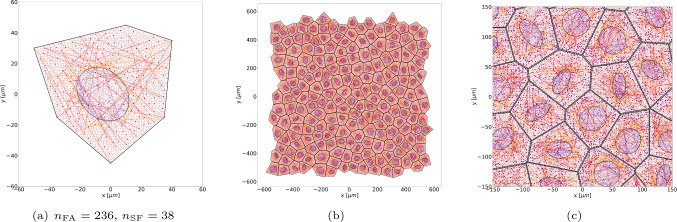
DNMs of cells and cell monolayers: (a) single EC with its number of FAs as well as SFs, (b) EML with 203 cells spanning approximately 1 mm^2^, and (c) close-up of the central EML region. The dark red network represents the actin cortex, red indicates ventral SFs, orange perinuclear SFs, blue intranuclear actin, black nuclear and cellular membranes, and purple dots represent FAs

An example of the corresponding EC DNM is shown in Fig 2a.

#### DNM of endothelial monolayers

The algorithm to generate single ECs was extended to generate DNMs of EMLs. To this end, a Voronoi mesh was created from random seed points. The *n* cells form the basis for *n* ECs. Copies of the vertices of each polygon were moved toward its centre by a small amount $\delta$. The thus generated disconnected polygons were used to generate cell DNMs according to Sec. 2.2.1, which were thereafter connected to each other at their membrane connection points (adherens junctions). An example of a DNM of a EML with $n=203$ cells is shown in Fig 2b,c.

#### Fibre properties

**Fig. 3:**
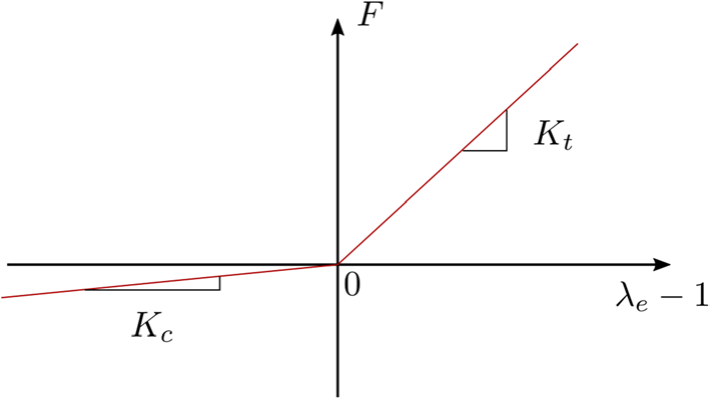
Bilinear material law with tensile and compressive stiffness $K_{\textrm{t}}$ and $K_{\textrm{c}}$

All fibres were considered elastic with a bilinear dependence of the force *F* on the elastic stretch $\lambda _{\textrm{e}}=r/r_{\textrm{a}}$, characterised by two moduli $K_{\textrm{t}}$ and $K_{\textrm{c}}$ in tension and compression, respectively (Fig. 3). Deguchi et al (2005) reported that ventral SFs are prestretched in homeostasis so that their length *r* depends on both their elastic stretch $\lambda _{\textrm{e}}$ and an activation parameter $a<1$. A multiplicative split (cf. Martins et al 2006) was assumed such that

1 $$\begin{aligned} {r} = \lambda _{\textrm{e}}\, a \,r_0 = \lambda _{\textrm{e}} \,r_{\textrm{a}}, \end{aligned}$$

where $r_{0}$ is the length of the fibre in the DNM as it was generated, while $r_{\textrm{a}} = a\, r_0$ corresponds to the shortened, zero-force state of an isolated activated fibre. For non-activated fibres ($a=1$) as well as non-actinous membranes, it follows $r_\textrm{a}=r_0$.

For ventral SFs, Deguchi et al (2005) determined a fibre stiffness of 20.2 nN and measured a fibre activation of $a_{\textrm{SF}}=0.81\pm 0.11$. Taking into account that the perinuclear SFs are inclined with respect to the representation plane, their effective stiffness was considered with only one-fourth of the ventral SFs (5.05 nN), and the activation was set to 0.9. For the actin filaments of the cortical actin, Kojima et al (1994) reported a spring constant of $(43.7\pm 4.6)$  nN/$\mu$m. Assuming an average filament length of $1.5\,\mu$m in this work, we adopted an average actin filament stiffness of 29.1 nN. Inspired by the procedure of Vignaud et al (2021), the cortical actin filament activation was set to a quarter of the ventral SF activation, which resulted in a value of 0.95. Since information on the compressive properties of the slender structures were generally missing, it was assumed that $K_{\textrm{c}}=K_{\textrm{t}}/10$, and parameters for cellular membrane, nuclear actin, and nuclear membrane were estimated as summarised in Table 2. The choice of parameters is further discussed in Sect 4.2.

**Table 2:** Tensile (t) and compressive (c) stiffnesses of fibre and membrane bonds, and activation *a* of actin components in the DNM

	$K_{\textrm{t}}$ [nN]	$K_{\textrm{c}}$ [nN]	*a* [−]
Actin cortex	29.1	2.91	0.95
Ventral SF	20.2	2.02	0.81
Perinuclear SF	5.05	0.51	0.9
Nuclear actin	1000	100	1
Cellular membrane	0.1	0.01	−
Nuclear membrane	1000	100	−

#### Interactions with a deformable substrate

Cells in 2D cell culture adhere to their substrates underneath at the FA complexes. The forces within the cell must, therefore, be in equilibrium with the forces acting on the substrate at the connection points (FAs). Due to either activation of the cytoskeletal fibres or external loads acting on the substrate, both the cell and the substrate may generally deform. If the substrate is rigid, the deformation is restricted to the cell and can be computed by solving the corresponding mechanical boundary value problem (BVP) with kinematic (displacement) boundary conditions (BC) set to zero at the FAs. If the substrate is deformable, these connection points move depending on the substrate’s mechanical properties until mechanical equilibrium of cell and substrate is reached. Therefore, the DNM and a continuous substrate model need to be coupled. The latter can be used in implicit finite element (FE) analysis with Newton-type solvers. Depending on the structure of the DNM, there exist more robust solvers for solving the DNM, such as the here proposed conjugate gradient implementation in LAMMPS (Thompson et al 2022). The FE models and DNMs were, therefore, solved independently and coupled by means of an iterative scheme.

### Solution of cell model BVP with LAMMPS

Imposing kinematic BCs on the FA points in the DNM, potentially while activating the stress fibres and the actin cortex, sets mechanical BVPs for the DNM, which were solved in LAMMPS (v. Dec. 2022). To simulate the DNM in the atomistic framework of LAMMPS, fibre connection points (nodes) were represented as atoms, and fibres were represented as covalent bonds. The mass of the atoms does not influence the result of the energy minimisation and therefore was arbitrarily set to 1 mg. While energetic potentials of covalent bonds depend on the distance between the two atoms forming the bond, in fibre networks, the strain energy of individual fibres typically depends on the fibre strain. For this reason, based on the existing distance-dependent bond potential *harmonic*, a new strain-dependent bond potential *bilinear* was defined as

2 $$\begin{aligned} E = \frac{1}{2}\left[ K_{\textrm{t}} \bigg \langle \frac{r-r_\textrm{a}}{r_{\textrm{a}}} \bigg \rangle ^2 + K_{\textrm{c}} \bigg \langle \frac{r_\textrm{a}-r}{r_{\textrm{a}}} \bigg \rangle ^2\right] , \end{aligned}$$

to reflect the tension-compression asymmetry of the fibres, where $\langle \rangle$ denote Macauley brackets.

The *fix setforce* command was used to impose displacement BCs on the FA points. Thereafter, the displacement of all nodes was computed by energy minimisation using the *conjugate gradient* method with the *forcezero* line search algorithm and a cumulative force threshold of 1 pN as criterion for convergence.

### Solution of substrate FE model with Abaqus

The substrate was represented as an elastic cuboid block with dimensions $1000\times 1000\times 100$ $\mu$m in Abaqus/Standard (v. 2021) with fixed lateral and bottom surfaces. Its central superficial region of $200\times 200\times 25$ $\mu$m was separately meshed with linear triangular prisms (C3D6H) and connected to the remaining part, meshed with linear tetrahedral elements (C3D4H), with tie surface constraints. Neo-Hookean hyperelastic material properties were used, with material parameters $C_{10} = 1.166$ kPa and $D_1 = 1.2008$ MPa^-1^, providing a Young’s modulus of $\sim\!7$ kPa in line with the substrates used by Reyes Lúa 2020. Each FA was represented by placing a reference node on the substrate’s surface at the coordinates of the FA nodes originating from the DNM formulation, and its region of impact was modelled as an ellipse with a major axis of 2 $\mu$m and minor axis of 1 $\mu$m around this reference node (Zündel et al 2017a). Moreover, the major axis of each FA’s elliptical region of impact was oriented in the direction of the load or displacement. This information was gathered from the solution of the cell model BVP with LAMMPS described in Section 2.3. Each FA domain was represented by approximately 30 nodes, whose displacements were subject to a kinematic coupling constraint, such that each FA deformed as a rigid body on the substrate surface. The mesh resolution in the depth of the central region was restricted to six elements with simple bias towards the top, resulting in a surface element depth of 1 $\mu$m. The mechanical BVP was then set up by imposing displacement BCs (or loads) on all FAs’ reference nodes. Subsequently, the reaction forces $\varvec{ f }_{\textrm{S}}$ corresponding to the displacements of these nodes were evaluated. The appropriate size of the FEs was chosen by studying convergence of the results in terms of the forces $\varvec{ f }_{\textrm{S}}$ for decreasing mesh sizes, thereby increasing the number of the elements (Suppl. Mat. A).

### Coupling of cell and substrate models

**Fig. 4:**
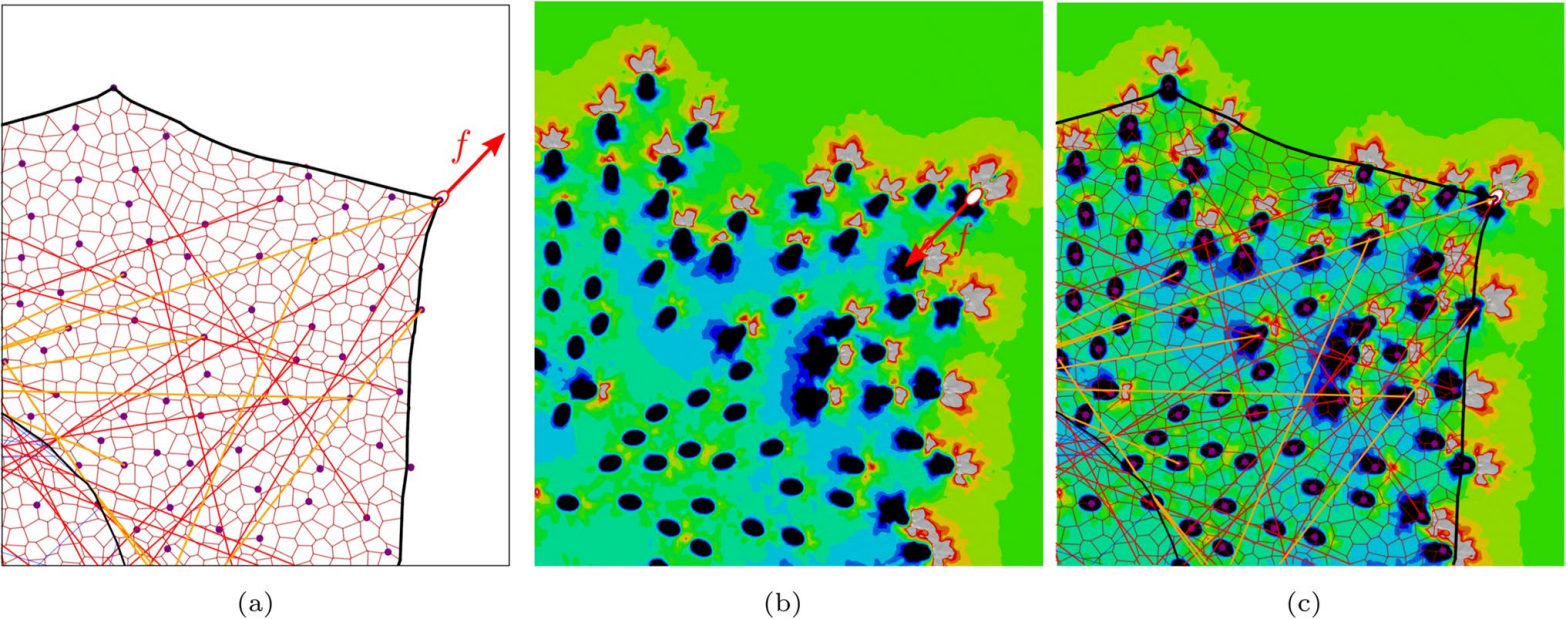
Schematic representation of the coupled cell and substrate models: The force at a single FA in the cell DNM (a) and the substrate FE model (b), and coupled system in equilibrium. Colours in (b,c) indicate local area strain (Eq. (5)); due to the representative character of the figure, scale bars were omitted

**Fig. 5:**
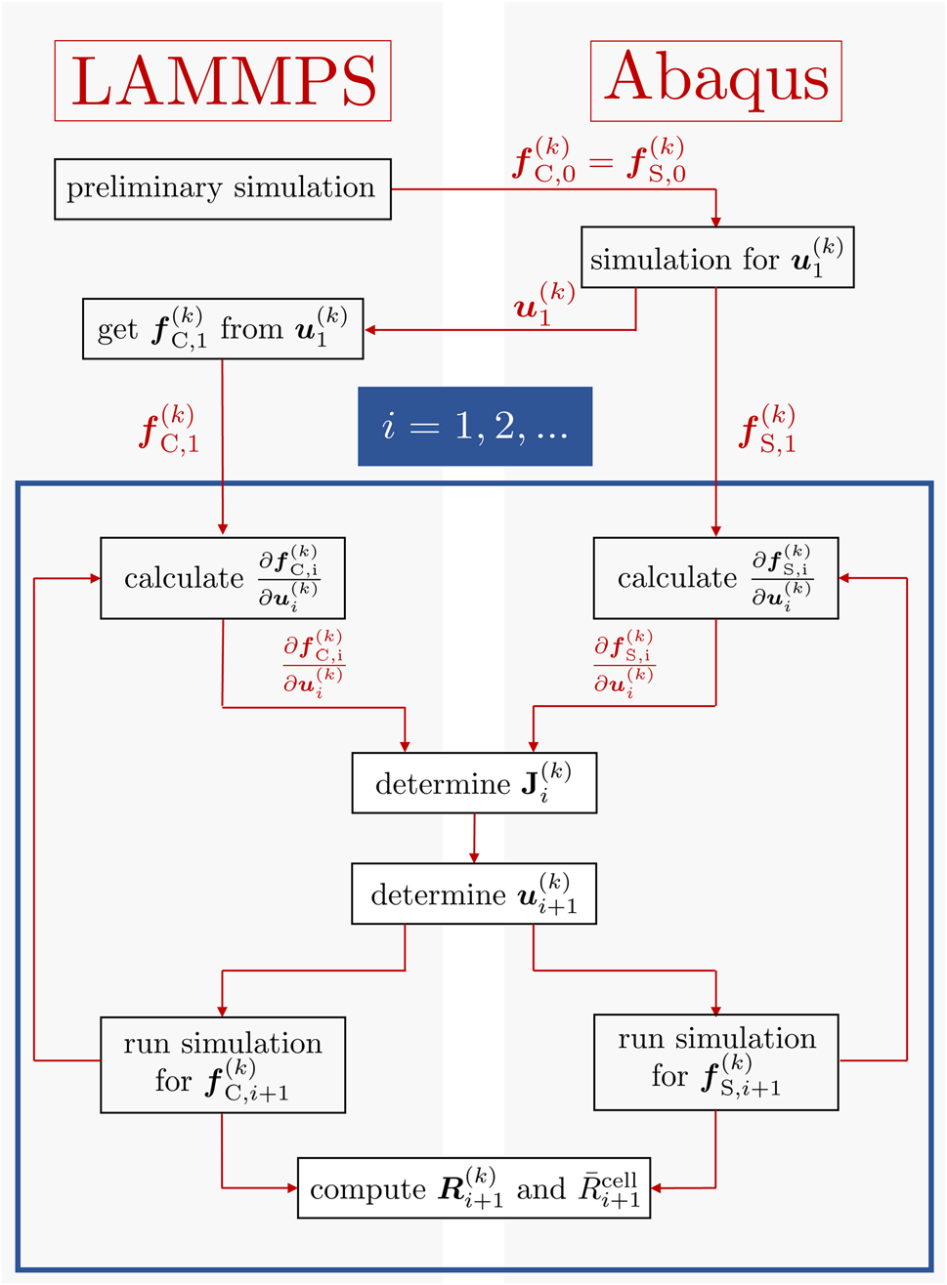
Graphical summary of numerical procedure for coupling between cell and substrate models (see Suppl. Mat. B)

Since cell DNM and the continuous substrate FE model were solved by different software, they were weakly coupled through a Python script by enforcing equal displacements $\varvec{ u }^{(k)}$ in both models at each individual FA site *k*. The equilibrium of forces requires that the corresponding reaction forces $\varvec{ f }_{\textrm{C}}$ computed with LAMMPS and $\varvec{ f }_{\textrm{S}}$ computed by Abaqus also coincide in magnitude, and have opposite sign (Fig. 4). The corresponding displacements $\varvec{ u }^{(k)}$ were determined by definition of the residuals $|\varvec{ R }^{(k)}|^2={|\varvec{ f }^{(k)}_{\textrm{S}}+\varvec{ f }^{(k)}_\textrm{C}}|^2$ at each FA, which were minimised by a numerical procedure. Given the computational cost associated with the FE analysis on a highly refined mesh, this procedure was simplified towards reducing the number of FE analyses and was based on a simplified Levenberg–Marquardt type algorithm (Chong and Žak 2008, Ch. 9) as described in full detail in Suppl. Mat. B. Figure 5 provides a graphical summary of the procedure. Briefly, it was assumed that in both the substrate and cell models the change of the displacement $\varvec{ u }^{(k)}$ of FA *k* has much stronger effect on the corresponding force $\varvec{ f }^{(k)}$ than those of the other FAs $j\ne k$, so that the influence of the latter could be neglected. With this assumption, the residual $\varvec{ R }^{(k)}$ for values close to a given $\varvec{ u }^{(k)}_*$ could be approximated by

3 $$\begin{aligned} \varvec{ R }^{(k)}(\varvec{ u }^{(k)}_*\!+\!\Delta \varvec{ u }^{(k)}) \approx \varvec{ R }^{(k)}(\varvec{ u }^{(k)}_*)+\!\left. \frac{\partial \varvec{ R }^{(k)}}{\partial \varvec{ u }^{(k)}}\right| _{\varvec{ u }^{(k)}_*}\!\!\!\!\!\!\!\Delta \varvec{ u }^{(k)}, \end{aligned}$$

which served to formulate an update rule for $\Delta \varvec{ u }^{(k)}$ to minimise $|\varvec{ R }^{(k)}|^2$. The four scalar components of the *k* simplified Jacobians ${\partial \varvec{ R }^{(k)}}/{\partial \varvec{ u }^{(k)}}$ were computed by finite difference approximations.

As an overall measure of the goodness of fit, a cell-scale residual was defined by taking the 2-norm of the FA specific residuals $|\varvec{ R }^{(k)}|^2$

4 $$\begin{aligned} {\bar{R}}^{\textrm{cell}} =\sqrt{\sum _{k=1}^{n} \left| \varvec{ R }^{(k)}\right| ^2}, \end{aligned}$$

which was evaluated in each step, and used as a criterion to stop the algorithm.

### Validation by analysis of cell-induced substrate strains

Traction force microscopy (TFM) is an established experimental method to quantify the forces exerted by individual cells on their substrate. By monitoring the cell-generated deformation of a flexible substrate with known material characteristics, the solution of the inverse problem serves to compute the cellular forces (Bergert et al 2016; Zancla et al 2022; Butler et al 2002). In this work, intermediate data from multistep TFM are considered in which the deformation fields are quantified for the reference state as well as after applying an external stretch to the flexible, nonlinear elastic substrate. The combination of the applied stretch with the locally induced cellular contraction leads to additional information about cytoskeletal mechanics such as the relationship between the cytoskeletal activation and substrate stiffness.

#### Benchmark: TFM on equibiaxially stretched substrates

Reyes Lúa (2020) conducted TFM with and without applying an equibiaxial substrate strain of $\varepsilon \approx 0.05$ corresponding to 10% area strain. These data will be used to benchmark the cell model presented herein.

The cells had been seeded on a soft silicone elastomer (Young’s modulus: $E\approx$7 kPa) equipped with fluorescent markers on the surface. Laser microscopy images were recorded in three different configurations: the substrate with cell and without externally applied load (0%), the substrate with the cell immediately after a 10% increase in membrane area by external loads, and the unstrained substrate after removal of the cell. The Fiji plugin bUnwarpJ that employs elastic image registration based on B-splines (Schindelin et al 2012; Arganda-Carreras et al 2006) was used to correlate the images in the deformed states (0% or 10%) with the undeformed control image based on a grid of 8x8 B-splines. The deformation field was derived from the interpolated planar displacement fields. Calculating the local in-plane principal stretches $\lambda _1$ and $\lambda _2$, Reyes Lúa (2020) defined the area strain as

5 $$\begin{aligned} \varepsilon _{\textrm{A}} =\lambda _1\lambda _2-1. \end{aligned}$$

The region underneath the cell and the region in the cell’s vicinity (approximately four times bigger) were specified, and the area strain values in these two regions were binned into four bins, respectively. The aggregated area strain underneath the cell was then calculated as the average of the two bins with lower values weighted by their respective occurrences. The aggregated area strain surrounding the cell was defined as the value of the bin with the highest occurrence in this region. The difference $\Delta \varepsilon _{\textrm{A}}$ of the two regions’ aggregated area strains was defined as a measure of cellular contraction on the substrate. Data from 31 HUVECs were considered for model validation. In addition, TFM experiments on human foreskin fibroblasts were used to validate a corresponding model of a single fibroblast (Suppl. Mat. C).

#### In silico replication of TFM experiments

To computationally replicate these TFM experiments, $n=5$ DNMs each representing a statistical realisation of a cell were generated according to Sect 2.2.1. To start the iterative minimisation scheme (Sec. 2.5), the FA nodes of the DNM were either fixed at their original positions $\varvec{ X }^{(k)}=\{X_1^{(k)},X_2^{(k)}\}$ leading to zero initial displacements

6 $$\begin{aligned} \varvec{ u }^{(k)}_0 = \varvec{ 0 } \quad \quad \text {(0\% substrate area strain)} \end{aligned}$$

or subjected to the displacements

7 $$\begin{aligned} \varvec{ u }^{(k)}_0 = \bigl (\sqrt{1.1}-1\bigr ) \varvec{ X }^{(k)}\quad \text {(10\% substrate area strain),} \end{aligned}$$

reflecting the case of 10% nominal area strain applied to the substrate.

To enable a direct comparison between experiment and simulation by applying the same image analysis techniques (Reyes Lúa 2020), subsets of the substrate surface nodes in the inner domain of the FE mesh were utilised to define strain markers analogous to the fluorescent markers in the experiments. The sets were created by randomly seeding points on the substrate surface and determining the mesh nodes closest to these points. The number of points was chosen such that their number roughly corresponded to the fluorescent dots in the experiments (Reyes Lúa 2020). Using the referential and deformed coordinates of these nodes, grey scale images were created with image resolution of $0.229\,{\mu\text{m/px}}$ placing at each node a marker with Gaussian intensity profile and 9 px diameter.

These images served as input for the image analysis described by Reyes Lúa 2020, i.e. images were correlated with bUnwarpJ, area strain information was calculated, and the area strain difference was determined in the same way as described in Sect 2.6.1.

To estimate whether the coarse (only $8\times 8$) grid used for bUnwarpJ’s B-spline interpolation would be sensitive to the random choice of virtual strain markers, the above procedure was repeated twice with different sets of substrate surface mesh nodes for the creation of the grey scale images (Suppl. Mat. D).

### Parameter study

Given the limited experimental information on cytoskeletal fibre properties, the sensitivity of the results to changes in the chosen mechanical parameters (Table 2) was studied by repetition of HUVEC simulations upon individually changing the most effective ones by 30%. The stiffness $K_{\textrm{t}}$ (and $K_\textrm{c}=0.1\,K_{\textrm{t}}$) of cortical actin and ventral SFs was increased by 30%, and their activation value *a* was decreased by 30$\%$, respectively (Table 3), in a one-at-a-time analysis.

Furthermore, to investigate the influence of the variability of mechanical properties within the cell due to, e.g. different fibre diameters, $K_{\textrm{t}}$ and $K_{\textrm{c}}$ were varied according to a normal distribution with a mean given by the control value and a coefficient of variation of 30%. In some rare cases when the stiffness due to this procedure turned negative, it was set to 0.

**Table 3:** Stiffness and activation parameters of cortical actin and ventral SFs for the different repetitions, based on the same $n=5$ HUVEC DNMs as used in the study. Repetitions 1-4 were based on deterministic values, 5 and 6 on normal distributions with CoV=STD/mean of 30%

	Control	rep. 1	rep. 2	rep. 3	rep. 4	rep. 5	rep. 6
$K_{\textrm{t}}$ cortex [nN]	29.1	37.8	29.1	29.1	29.1	$29.1\pm 8.73$	29.1
*a* cortex [−]	0.95	0.95	0.935	0.95	0.95	0.95	0.95
$K_{\textrm{t}}$ ventral SF [nN]	20.2	20.2	20.2	26.3	20.2	20.2	20.2±6.06
*a* ventral SF [−]	0.81	0.81	0.81	0.81	0.75	0.81	0.81

## Results

### Energy minimisation in LAMMPS

**Fig. 6:**
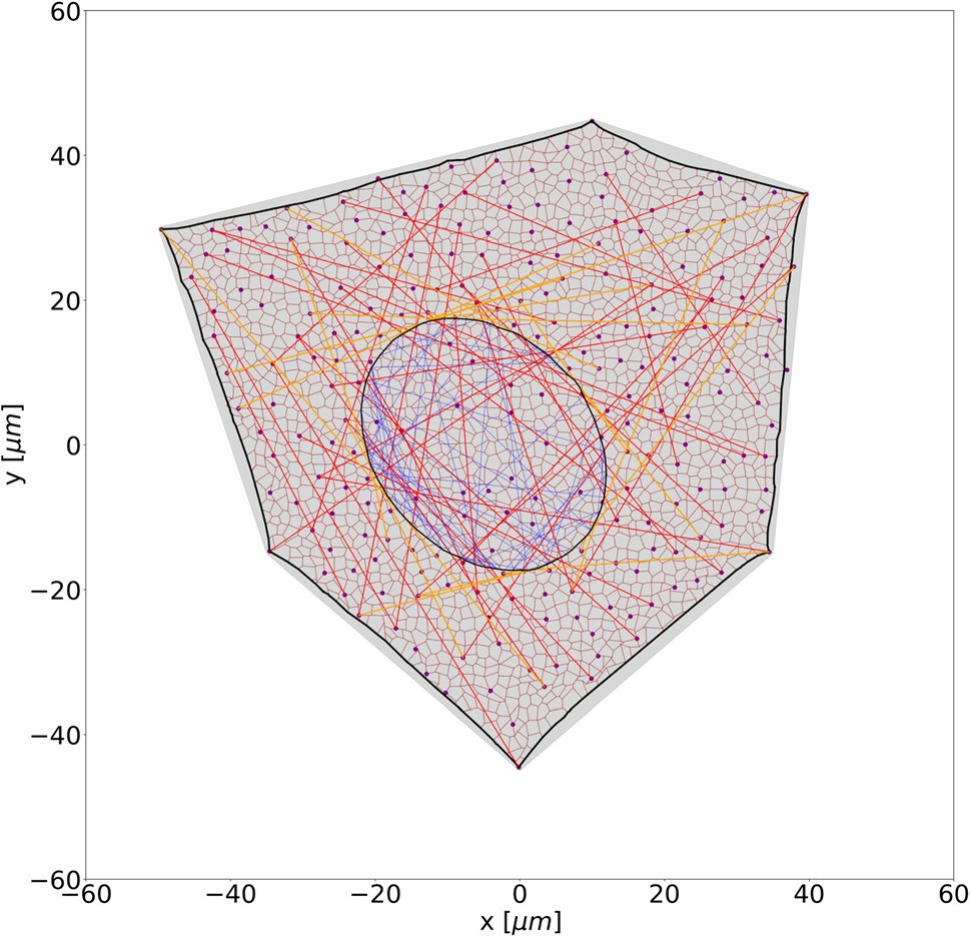
Minimal energy configuration of the DNM depicted in Fig 2a at 0% substrate area strain. For comparison, the grey area denotes the domain occupied by the DNM in reference configuration

Constraining the FA nodes of the cell models (Fig. 2a) by the displacements $\varvec{ u }^{(k)}_0$ according to Eq. (6) or (7) and changing the activation parameter from 1 to the values specified in Tabs. 2 and 3, the corresponding BVPs were set-up and solved with LAMMPS by energy minimisation.

Exemplary results for a HUVEC-type cell are shown in Fig 6 for $\varvec{ u }^{(k)} = \varvec{0}$ (6). Note the slight change in shape compared to the reference state (shaded in grey), most prominently recognised in the curved shape of the cell membrane.

### In silico replication of TFM experiments

#### Coupling of DNM and FE analysis

**Table 4:** ${\bar{R}}^{\textrm{cell}}$ (4) in units of nN for the HUVEC models under zero and 10% substrate area strain

Subst. area strain	${0\%}$	${10\%}$
Cell 1	0.98	1.66
Cell 2	1.35	1.92
Cell 3	1.14	2.07
Cell 4	1.20	1.97
Cell 5	1.56	1.87
Mean	1.25	1.90

**Fig. 7:**
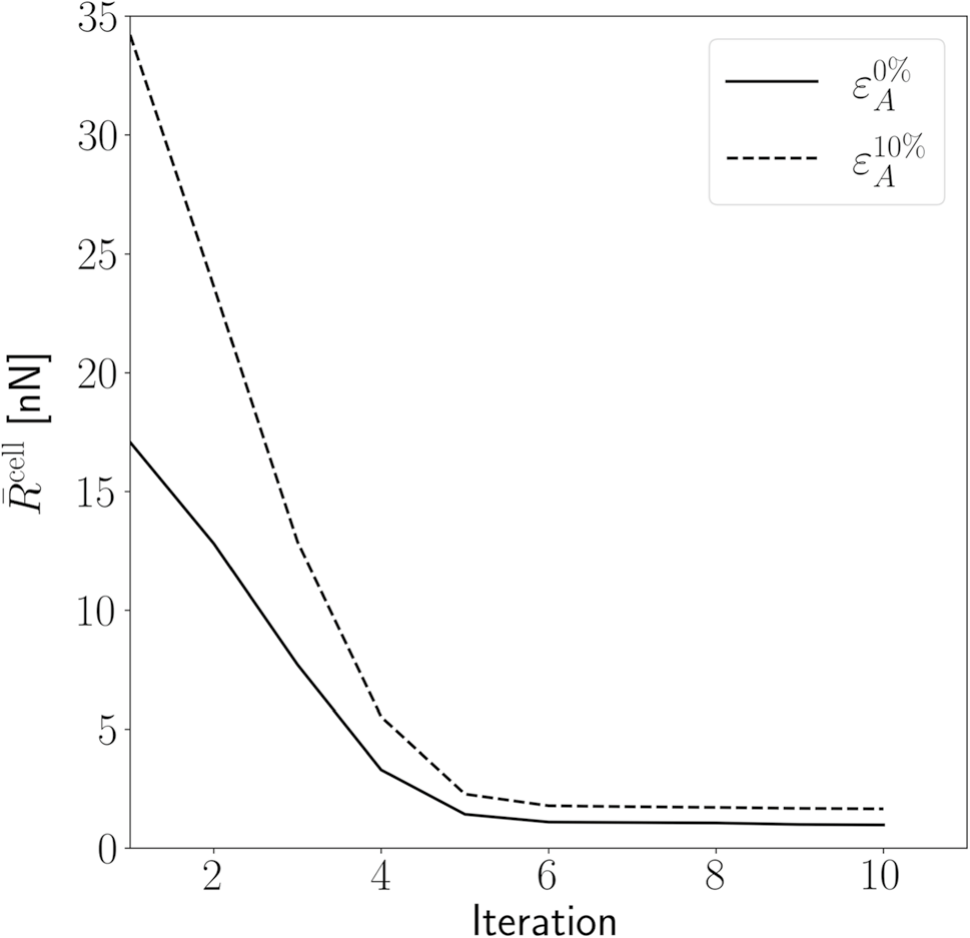
Cell-scale residual ${{\bar{R}}}^{\textrm{cell}}$ (4) for one of the simulated cells

To take into account the inhomogeneous deformation field generated by the cells residing on a soft, deformable, substrate, the DNM and FE model were coupled according to Sect 2.5. The cell-scale residual (4) is given for the $n=5$ cells analysed in Table 4. Moreover, Fig 7 shows the reduction of the residual ${\bar{R}}^{\textrm{cell}}$ with the first 10 iterations of the coupling algorithm for one cell. A closer analysis of the distribution of the single-cell residuals $|\varvec{ R }_i^{(k)}|$ reveals that the remaining residual results from a few FAs with remaining high error, while the vast majority of FAs has small residuals (Suppl. Figure S2). This behaviour was similar for all $n=5$ cells, and ${\bar{R}}^{\textrm{cell}}$ was reduced to 7.4% of its initial values for the unstretched, and to 5.5% for the 10% area strain case within the 10 steps.

#### Local area strain in the substrate

**Fig. 8:**
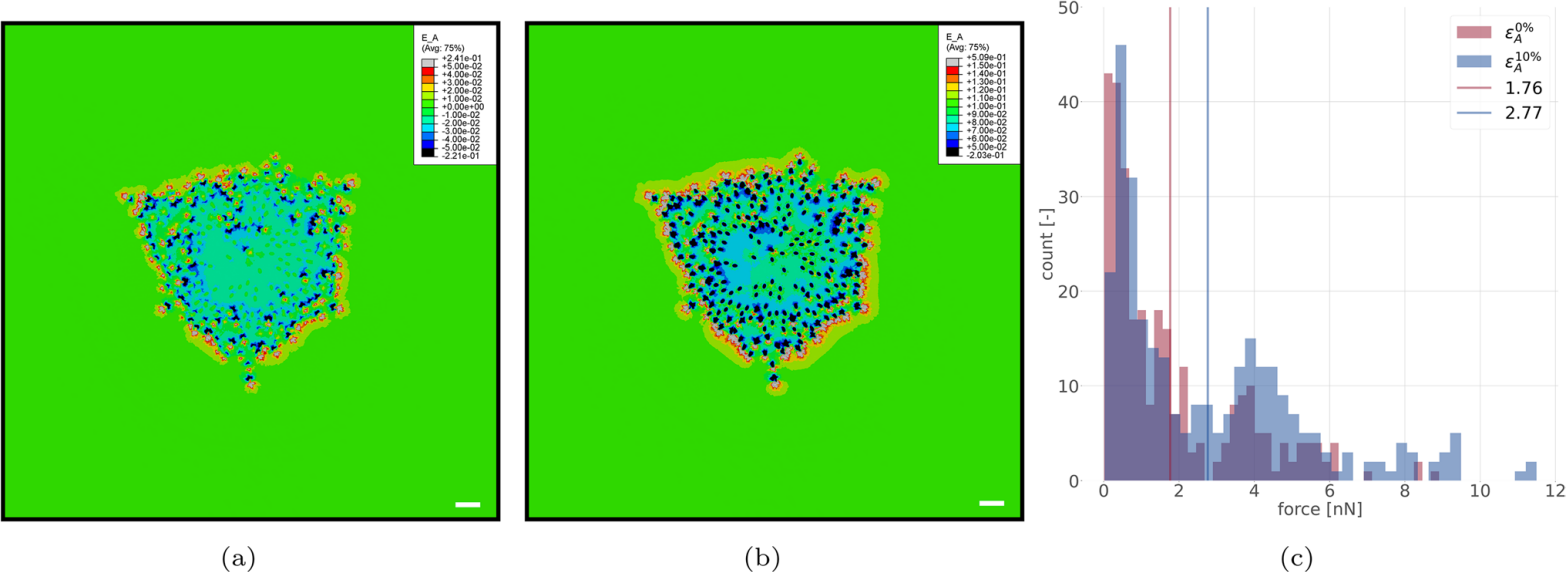
FA forces and area strains on the substrate: (a) and (b) computed local area strain field $\varepsilon _{\textrm{A}}$ (5) for either 0% (a) or 10% (b) overall substrate area strain. Scale bar: 10 $\mu$m. (c) Distribution of FA forces for the two load cases. Vertical bars denote their respective mean values

Due to the interaction with the cell, the local substrate area strain $\varepsilon _{\textrm{A}}$ according to Eq. (5) differs from the applied overall area strain of 0% and 10% achieved by displacing the substrate boundaries. Using the nominal strain field computed by the FE analysis in the accepted 10th iteration of the optimisation algorithm, $\varepsilon _{\textrm{A}}$ (5) was computed from the in-plane principal strains (Fig. 8).

#### Distribution of traction forces

Figure 8c shows a histogram of the corresponding traction forces acting on the substrate. One notes a shift of the mean traction force when increasing $\varepsilon _\textrm{A}$ from 0 to 10 %. Noteworthy, the increased FA forces are a direct consequence of the increased tension in the linear elastic fibres representing the cytoskeleton. They are hence a purely passive mechanical phenomenon and do not require active cellular processes such as cytoskeletal stiffening (Mann et al 2012) or contraction reinforcement (Krishnan et al 2009).

#### Image analysis of the virtual experiments

**Fig. 9:**
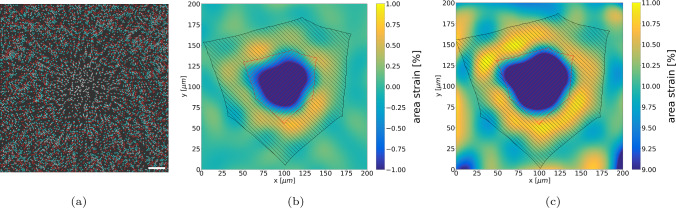
In silico replication of TFM method by Reyes Lúa (2020): (a) overlay of virtual displacement markers in the reference state (cyan) and upon contraction of a HUVEC cell on a substrate with 10% overall substrate strain (red). Note that overlapping markers appear in white. Scale bar: 20$\mu$m. (b) Local area strain after DIC with bUnwarpJ for 0% and (c) 10% overall substrate strain. Hatching in red and black indicates underneath-cell and cell-vicinity areas, respectively

The coupled DNM-FE simulations were treated as virtual TFM experiments and analysed analogously to the method presented in Reyes Lúa 2020. To this end, the substrate area strains were recomputed based on DIC with Fiji (Sec. 2.6.2). The sets of 9 px sized dots generated from nodes of the FE mesh used as virtual strain markers to be interpolated with bUnwarpJ are shown in Fig 9a as an overlay plot: As marker deformations in the 0% global area strain case are barely visible, only the 10% case is shown here. The area strains based on the interpolated displacement fields reveal clear differences between the areas below the cell, in its vicinity and distant to it (Fig. 9b and c).

**Fig. 10:**
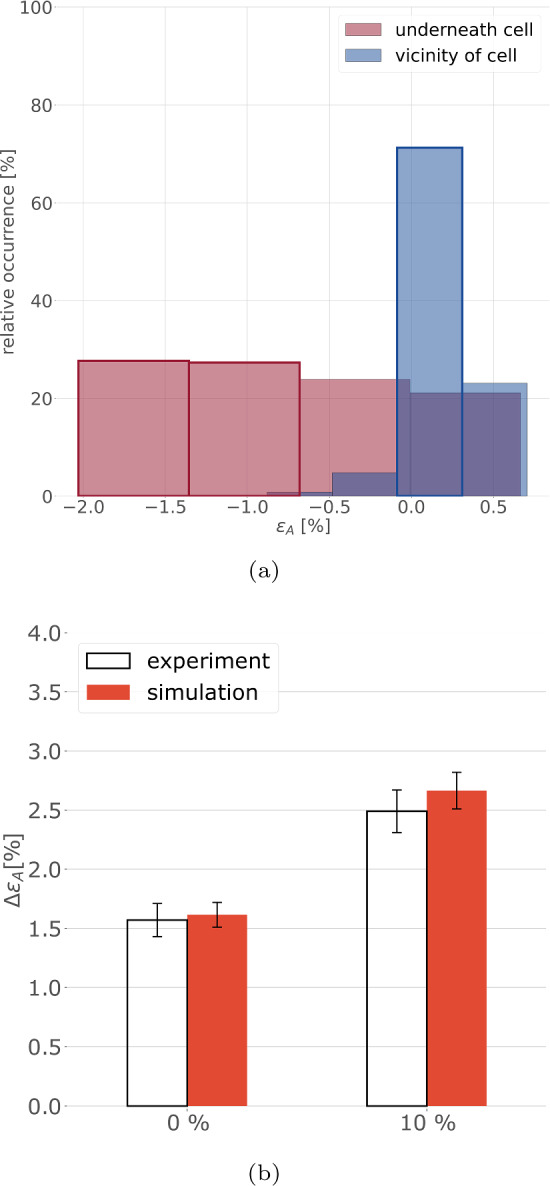
Evaluation of the area strain difference. (a) Normalised histograms of binned pixel-wise area strain obtained from the hatched regions in Fig 9b for 0% global area strain. Framed bins were used for the evaluation described in Sect 2.6.1. (b) Comparison of $\Delta \varepsilon _{\textrm{A}}$ with experimental data for 0% and 10% global area strain

### Area strain difference

The pixel-wise local area strains in Fig 9b and c were collected into four bins according to Reyes Lúa (2020), and the normalised histograms (Fig. 10a) were used to compute the difference $\Delta \varepsilon _{\textrm{A}}$ characterising the difference between strains underneath and around the cell, respectively (Fig. 10b). $\Delta \varepsilon _{\textrm{A}}$ was computed for $n=5$ HUVECs (Table 5). The corresponding comparison with the experimental values (Reyes Lúa 2020) in Fig 10b shows sound agreement.

**Table 5:** Results for area strain difference $\Delta \varepsilon _{\textrm{A}}$ for five HUVECs for the two load cases

	0% $[\%]$	10% $[\%]$
Cell 1	1.26	2.23
Cell 2	1.80	2.81
Cell 3	1.63	2.27
Cell 4	1.92	3.01
Cell 5	1.47	3.01
mean	1.62	2.66

### Sensitivity to changes in parameters

Given that many of the model parameters had to be estimated due to a lack of experimental information, we performed a one-at-a-time sensitivity analysis with respect to four parameters that have particularly strong effect on the results in terms of $\Delta \varepsilon _{\textrm{A}}$.

**Fig. 11:**
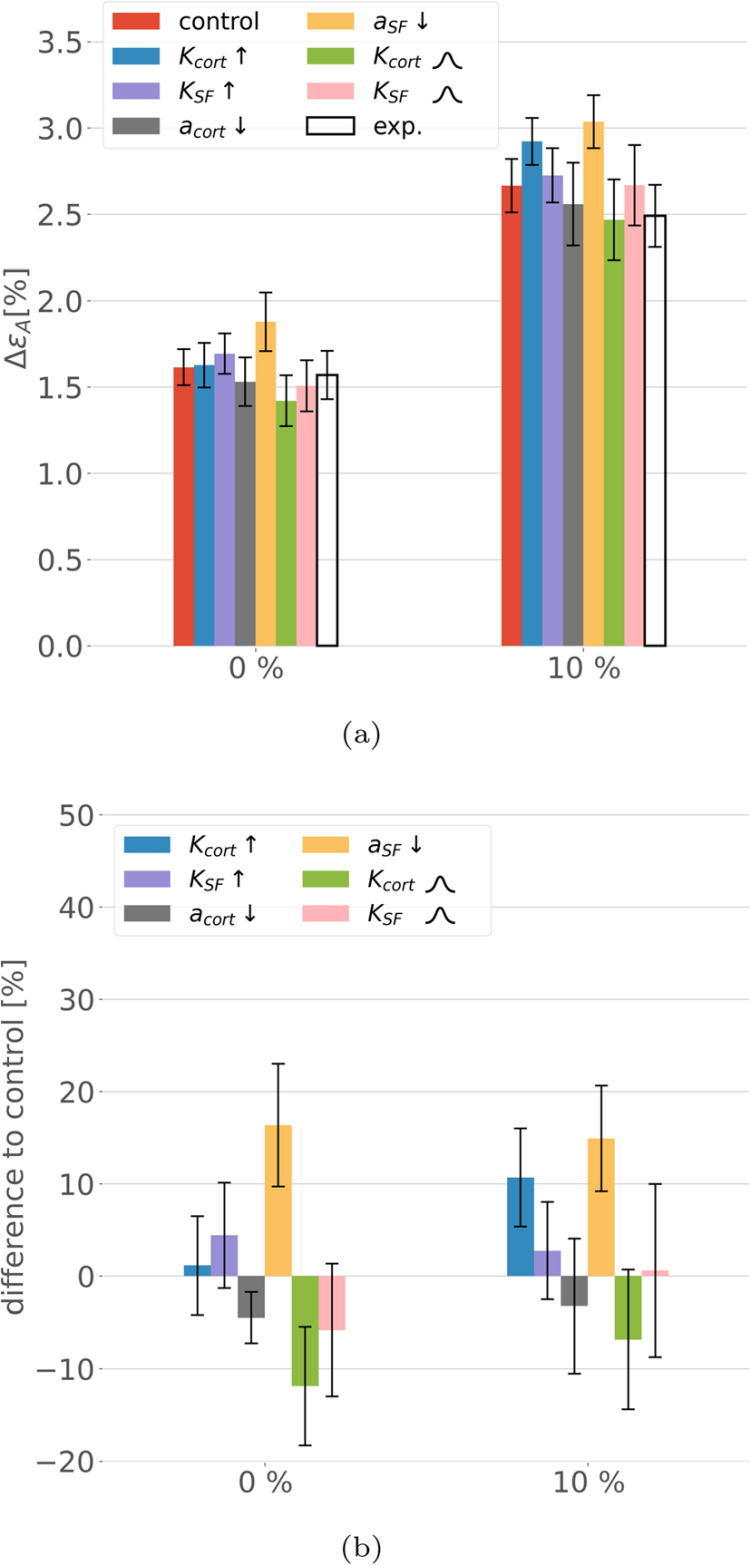
Sensitivity analysis for the stiffness and activation parameters of the cortex and ventral SFs, respectively.The bell curve symbol indicates a normal ($n=5$, mean±SEM). (a) Absolute values of the resulting area strains. (b) Relative difference to the control case. $\uparrow$ and $\downarrow$ indicate increase and decrease in the specific parameter by 30%, respectively. The small bell-shaped symbol indicates a normal distribution of the parameter around the given value with a CoV of 30%

The results (Fig. 11) show that the reduction of the SF activation by 30% has the strongest effect on the unstretched substrate as well as in the 10% substrate strain case. Nevertheless, it leads to corresponding changes in the response of less than 30%. In the 10% substrate strain case, an increase in the tensile (and accordingly compressive) stiffness of the cortical actin leads to changes comparable to the reduction of the SF activation. The more pronounced effect of $K_{\textrm{cort}}\uparrow$ on the 10% case can be explained by the dense, cell-spanning, and relatively regular cortex network effectively transferring the fibre stiffness to the cell-scale behaviour.

A probabilistic description of the network parameters led to generally smaller area changes $\Delta \varepsilon _{\textrm{A}}$ induced by the cell and hence indicate a softer response of the cell.

## Discussion

### Simplified representation of the endothelial cytoskeleton

The cytoskeleton is a complex, generally three-dimensional multi-component network connected to the cell and nuclear membranes as well as its surrounding. With the goal to address research questions associated with the behaviour and integrity of the endothelial cell monolayer as well as TFM on flat substrates, simplifications were considered as follows:

At first, we focused on representing the endothelial cytoskeleton as a two-dimensional random fibre network, projecting the most relevant components, including the nuclear envelope, onto the plane of the cellular floor.

Next, the cytoskeletal representation was restricted to the actin components, due to the minor role of IFs and MTs expected in the load states considered. Although it was suggested that the actin and IF networks indeed form an interwoven structure interacting at different length scales (Wu et al 2022), IFs appear slack in the homeostatic EC suggesting a low effective stiffness. Their contribution to the mechanical response is thus expected to be much smaller than that of the actin filaments under the moderate strains considered here.

Noteworthy, recent experimental and computational work on epithelial cells (Pensalfini et al 2023; Latorre et al 2018) pointed at an important role of the IF network and its reorganisation over long time spans in superstretched epithelial cells, i.e. in the high stretch regime. Also, MTs were shown to provide rigidity (Zhang and Guan 2020). Nevertheless, they were assumed to contribute mainly to the cell’s compressive properties according to the tensegrity model (Ingber 2003), which are of little relevance for the load states considered herein. The relatively low abundance of MTs compared to the other cytoskeletal filaments (Janmey et al 1991) further supports their omission in the model. Moreover, dorsal stress fibres and the transverse arcs connecting them were excluded, since the dorsal SFs are inclined out-of-plane so that their projections onto the plane of representation was assumed negligible.

While the distribution of FAs was presently modelled as fairly uniform with somewhat lower density underneath the nucleus in line with Nair et al (2021), recent investigations by Chala et al (2021) revealed a densification of FAs at the cell periphery. We remark that such or other spatial distributions could clearly be implemented by means of the current strategy for model generation. Moreover, the general framework would also serve to include the thus far omitted components of the cell, particularly intermediate filaments and microtubules, and likewise enable a 3D representation of the cell.

Finally, due to missing information about cell deformation in 3D and the reduction of the problem to 2D, we omitted a representation of the cytoplasm.

Despite these limitations, we consider the simplified model sufficient to capture the characteristic behaviour of ECs in the here addressed load cases. In addition, we remark that the availability of experimental data to characterise the mechanical properties of the single cytoskeletal components is strongly limited to date. Any additional component therefore would increase the list of unknown parameters in the model. Consequently, the present, simplified model represents a compromise between adding the relevant complexity and keeping the number of parameters with missing experimental support in a manageable range.

### Parametrising the cytoskeletal elements

The experimental data-based mechanical properties used in the simulations are limited to the tensile properties of ventral SFs and cortical actin. The lengths of fibrils in the cortex vary significantly with averages reported from 0.5 $\mu$m to 3 $\mu$m (McGrath et al 2000; Fritzsche et al 2016). The present choice of 1.5 $\mu$m is within this range, and together with the mean spring constant of 43.7 nN/$\mu$m (Kojima et al 1994), this leads to a filament stiffness of 29.1 nN. Vignaud et al (2021) showed that not only SFs but also cortical actin are activated in the homeostatic cell. In their investigation, they activated the cortex by one-fourth of the SFs. By adopting this approach, we accordingly reduced the active shortening, leading to $a=0.95$.

The values of the remaining parameters had to be estimated based on assumptions. Perinuclear SFs were assumed to have the same mechanical properties as the ventral SFs. However, because they span over the nucleus, they are not entirely parallel to the plane of the cellular floor. Therefore, they can rotate out of the plane of reference. Their effective contribution to the in-plane properties was hence reduced to $K_{\textrm{t}}=5.05$ nN and $a=0.9$, respectively. The membrane’s contribution to the cellular state of stress was assumed orders of magnitude smaller than the one of the actin network. It was included mainly for tracing cell boundaries, and the stiffness was arbitrarily set to 0.1 nN. Moreover, as intranuclear processes were not considered in this work, the nucleus was modelled as a quasi-rigid body within the cell, thus motivating very high (pseudo-)stiffness of nuclear membrane and nuclear actin ($K_{\textrm{t}}=1\,\mu$N).

Finally, the number of SFs within a HUVEC was utilised as an additional parameter to adjust the model response. Preliminary calculations revealed that for amounts of ventral SFs between 35 and 40 and perinuclear SFs between 10 and 20 the results fell into the range of the experimental results.

Despite the coarse estimates for these parameters, the sound quantitative agreement with the area strain observed in TFM experiments suggests that the parameters serve to adequately represent the contractile cell behaviour. Moreover, the performed sensitivity analysis (Sec. 3.4) underlines this statement. Particularly, $K_{\textrm{cort}}$, $K_{\textrm{SF}}$, and $a_\textrm{SF}$ seem to be chosen in a meaningful range as corresponding changes of 30% would worsen the agreement, while reducing $a_\textrm{cort}$ could only slightly improve the results.

### Computational methods to solve the BVPs

Typically, interactions between cells and deformable continuous substrates have been modelled by means of FE software (Dabagh et al 2017; Banerjee et al 2021). Solving large DNMs with implicit FE codes, however, can become cumbersome since the random connection between pin-jointed truss-like fibres can lead to sub-isostatic networks (Picu and Ganghoffer 2020, Ch. 1) and local instabilities. Therefore, minimising the potential energy by Newton-type solvers is prone to fail. Although stabilisation by local (Mauri et al 2016; Bircher et al 2017) or global (Zündel et al 2017b) damping, or the use of explicit FE codes (Domaschke et al 2019) or change of solver type may help to attenuate these problems, there exist more efficient techniques to minimise the potential energy of such large, locally interacting systems. MD software such as LAMMPS is particularly capable of addressing such problems when the cross-links are interpreted as ‘atoms’ and the fibres as ‘bonds’ between them. By using openMP and openMPI, LAMMPS employs shared memory multiprocessing and implements the message passing interface standard for high computational efficiency (Cha 2014). In addition, implementations of Hessian-free solvers such as conjugate gradient-based algorithms or FIRE (Guénolé et al 2020), which are frequently used for energy minimisation in sparse random fibre networks (e.g. Ruiz-Franco et al 2023) are directly available in LAMMPS.

While LAMMPS used with the conjugate gradient solver turned out to be a very robust choice for solving the DNM part of the problem, it was not applied to solve the boundary value problem for the continuous substrate part. On the one hand, the millimetre sized substrate domain precluded modelling the single molecular chains, i.e. to use MD in its original sense. On the other hand, ‘peridynamics’ approaches (Silling 2000) aim at using MD software for continuous mechanics problems. However, they require additional efforts to formulate peridynamic approximations of the here used hyperelastic incompressible constitutive law for the elastomeric substrate. For this reason, the BVP was solved separately for each of the domains by the most suitable technique, respectively. The two BVPs were then weakly coupled by the condition of force equilibrium at the FA nodes, where the cell and substrate domains were coupled kinematically.

The coupling algorithm was obtained by simplification of a Levenberg-Marquardt method to minimise the mean square of the residual forces. These simplifications aimed at reducing the number of evaluations of the FE model, which were associated with high computational times. Despite these approximations, the objective function had reduced to an acceptable value already after 10 iterations, and more than 60% of the remaining error originated from the residuals at less than 5% of the FAs. It is expected that a further optimisation of the parameters controlling the algorithm would serve to further reduce this error.

### Comparison with TFM experiments and limitations

Finally, as a first validation of the modelling approach, we compared the simulations with existing TFM data (Reyes Lúa 2020) in terms of the change of area strain $\Delta \varepsilon _{\textrm{A}}$ as it was defined in the experimental study.

To maintain the analysis workflow as close as possible to these experiments, the simulation outcome in terms of the computed displacement field was used to generate virtual images. These images were then processed by the same image analysis as used in Reyes Lúa 2020. As a consequence of adopting this technique, the determination of $\Delta \varepsilon _{\textrm{A}}$ that was compared with the experimental data is subject to the same limitations as in the original work (Reyes Lúa 2020). This mainly concerns the DIC method based on a B-spline interpolation on a grid of only $8\times 8$ B-splines with bUnwarpJ. Although we observed that the method is robust against the random choice of markers used for strain analysis, (Suppl. Mat. D.), the coarse interpolation might clearly mask details of the actual deformation field. This can also be detected when comparing the area strain field as obtained from the FE analysis (Fig. 8) with the interpolation result (Fig. 9).

Moreover, although the observed match with the experiments is very good, we critically remark that the agreement of the highly nonlinear model with these complex experiments in terms of the single parameter $\Delta \varepsilon _{\textrm{A}}$ can only provide an indication of the goodness of fit. This does by no means imply that all parameters are reliably determined, and all mechanisms within the cytoskeleton are captured correctly by the model. However, with regard to the magnitude of forces that the cell applies to its surrounding, the cell model may indeed provide a representative response. Thus, it qualifies for the use in computational studies on mechanical cell interactions within EMLs.

### Modelling EMLs

Section 2.2.2 describes how to generate models of many ECs connected to form an EML, and all computational procedures exemplified for single ECs likewise apply to EML models. Hence after the parametrisation of the cellular component models from TFM data as reported herein, the only ingredient required to perform corresponding simulations is a law that governs the properties of the connections between the cells, i.e. a corresponding *bond* type able to represent the properties of tight and adherens junctions between the cells.

## Conclusions

In this contribution, we proposed techniques to model single endothelial cells and endothelial monolayers with interconnected cytoskeleton as large discrete fibre networks. The molecular dynamics code LAMMPS was shown to be well-suited to solve mechanical boundary value problems that help understanding the mechanobiological in situ conditions of the cells. The model was used to simulate TFM experiments on single endothelial cells and fibroblasts on soft elastomeric substrates. To efficiently model this continuous material interacting with the cell, the substrate was simulated in FE software and the two models—discrete and continuous—were coupled by minimising the residual of the reaction forces at the contact points. The simulation results were analysed in full analogy with the existing experiments in terms of the local substrate area strains $\varepsilon _{\textrm{A}}$ induced by the contracting cells. The comparison revealed sound agreement, despite the many limitations of the model, mainly with regard to the choice of material parameters, which could only partly be specified by the literature values. The sensitivity of $\varepsilon _{\textrm{A}}$ to the choice of parameters obtained in a parameter study, however, suggests that the selected values are a meaningful choice to represent the mechanical properties of endothelial cells in the regime of loads investigated. Altogether, in addition to providing a computational tool to interpret TFM experiments, the here established methods form the basis for studying the mechanics of endothelial monolayers under various loading conditions for cardiovascular physiology and pathology.

### Supplementary Information

Below is the link to the electronic supplementary material.

Supplementary file 1 (pdf 5893 KB)

## References

[CR1] Alamé G, Brassart L (2019). Relative contributions of chain density and topology to the elasticity of two-dimensional polymer networks. Soft Matter.

[CR2] Amos SE, Choi YS (2021). The cancer microenvironment: Mechanical challenges of the metastatic cascade. Frontiers in Bioengineering and Biotechnology.

[CR3] Arganda-Carreras I, Sorzano COS, Marabini R, et al (2006) Consistent and elastic registration of histological sections using vector-spline regularization. In: Beichel RR, Sonka M (eds) Computer Vision Approaches to Medical Image Analysis. Springer Berlin Heidelberg, pp 85–95, 10.1007/11889762_8

[CR4] Autar A, Taha A, van Duin R (2020). Endovascular procedures cause transient endothelial injury but do not disrupt mature neointima in Drug Eluting Stents. Scientific Reports.

[CR5] Banerjee A, Khan MP, Barui A (2021). Finite element analysis of the influence of cyclic strain on cells anchored to substrates with varying properties. Medical and Biological Engineering and Computing.

[CR6] Bansod YD, Matsumoto T, Nagayama K (2018). A finite element bendo-tensegrity model of eukaryotic cell. Journal of Biomechanical Engineering.

[CR7] Barreto S, Clausen CH, Perrault CM (2013). A multi-structural single cell model of force-induced interactions of cytoskeletal components. Biomaterials.

[CR8] Barton DL, Henkes S, Weijer CJ (2017). Active Vertex Model for cell-resolution description of epithelial tissue mechanics. PLoS Computational Biology.

[CR9] Belmonte JM, Leptin M, Nédélec F (2017) A theory that predicts behaviors of disordered cytoskeletal networks. Molecular Systems Biology 13(9):941. 10.15252/msb.20177796 10.15252/msb.20177796 PMC5615920 28954810

[CR10] Bergert M, Lendenmann T, Zündel M (2016). Confocal reference free traction force microscopy. Nature Communications.

[CR11] Bircher K, Ehret AE, Mazza E (2017). Microstructure based prediction of the deformation behavior of soft collagenous membranes. Soft Matter.

[CR12] van Bodegraven EJ, Etienne-Manneville S (2021). Intermediate filaments from tissue integrity to single molecule mechanics. Cells.

[CR13] Bridson R (2007) Fast Poisson disk sampling in arbitrary dimensions. In: ACM SIGGRAPH 2007 sketches, pp 22–es, 10.1145/1278780.1278807

[CR14] Britt BR, Ehret AE (2022) Constitutive modelling of fibre networks with stretch distributions. part i: Theory and illustration. Journal of the Mechanics and Physics of Solids 167:104960. 10.1016/j.jmps.2022.104960

[CR15] Broussard JA, Jaiganesh A, Zarkoob H, et al (2020) Scaling up single-cell mechanics to multicellular tissues - the role of the intermediate filament-desmosome network. Journal of Cell Science 133:jcs228031. 10.1242/JCS.228031 10.1242/jcs.228031 PMC7097224 32179593

[CR16] Burridge K, Guilluy C (2016). Focal adhesions, stress fibers and mechanical tension. Experimental Cell Research.

[CR17] Butler JP, Tolic IM, Fabry B (2002). Traction fields, moments, and strain energy that cells exert on their surroundings. Am J Physiol Cell Physiol.

[CR18] Cha K (2014) Performance evaluation of LAMMPS on multi-core systems. In: Proceedings - 2013 IEEE International Conference on High Performance Computing and Communications, HPCC 2013 and 2013 IEEE International Conference on Embedded and Ubiquitous Computing, EUC 2013, pp 812–819, 10.1109/HPCC.and.EUC.2013.117

[CR19] Chala N, Moimas S, Giampietro C (2021). Mechanical fingerprint of senescence in endothelial cells. Nano Letters.

[CR20] Chong EKP, Žak SH (2008). An Introduction to Optimization. John Wiley & Sons Ltd, Hoboken, NJ,.

[CR21] Chugh P, Paluch EK (2018) The actin cortex at a glance. Journal of Cell Science 131:jcs186254. 10.1242/jcs.186254 10.1242/jcs.186254 PMC6080608 30026344

[CR22] Dabagh M, Jalali P, Butler PJ (2017). Mechanotransmission in endothelial cells subjected to oscillatory and multi-directional shear flow. Journal of the Royal Society Interface.

[CR23] Das A, Sastry S, Bi D (2021). Controlled neighbor exchanges drive glassy behavior, intermittency, and cell streaming in epithelial tissues. Physical Review X.

[CR24] Deguchi S, Ohashi T, Sato M (2005). Evaluation of tension in actin bundle of endothelial cells based on preexisting strain and tensile properties measurements. MCB Molecular and Cellular Biomechanics.

[CR25] Dogterom M, Koenderink GH (2019). Actin-microtubule crosstalk in cell biology. Nature Reviews Molecular Cell Biology.

[CR26] Domaschke S, Zündel M, Mazza E (2019). A 3D computational model of electrospun networks and its application to inform a reduced modelling approach. International Journal of Solids and Structures.

[CR27] Escribano J, Chen MB, Moeendarbary E (2019). Balance of mechanical forces drives endothelial gap formation and may facilitate cancer and immune-cell extravasation. PLoS Computational Biology.

[CR28] Fang Y, Lai KWC (2016). Modeling the mechanics of cells in the cell-spreading process driven by traction forces. Physical Review E.

[CR29] Freedman SL, Banerjee S, Hocky GM (2017). A versatile framework for simulating the dynamic mechanical structure of cytoskeletal networks. Biophysical Journal.

[CR30] Fritzsche M, Erlenkämper C, Moeendarbary E (2016). Actin kinetics shapes cortical network structure and mechanics. Science Advances.

[CR31] Guénolé J, Nöhring WG, Vaid A (2020). Assessment and optimization of the fast inertial relaxation engine (FIRE) for energy minimization in atomistic simulations and its implementation in LAMMPS. Computational Materials Science.

[CR32] Hamester F, Stürken C, Saygi C (2022). Insights into the steps of breast cancer-brain metastases development: Tumor cell interactions with the blood-brain barrier. International Journal of Molecular Sciences.

[CR33] Hartsock A, Nelson WJ (2008). Adherens and tight junctions: Structure, function and connections to the actin cytoskeleton. Biochimica et Biophysica Acta - Biomembranes.

[CR34] Hu J, Li Y, Hao Y (2019). High stretchability, strength, and toughness of living cells enabled by hyperelastic vimentin intermediate filaments. Proceedings of the National Academy of Sciences of the United States of America.

[CR35] Ingber DE (2003). Tensegrity i. cell structure and hierarchical systems biology. Journal of Cell Science.

[CR36] Jakka VVSV, Bursa J (2021). Finite element simulations of mechanical behaviour of endothelial cells. BioMed Research International.

[CR37] Janmey PA, Euteneuer U, Traub P (1991). Viscoelastic properties of vimentin compared with other filamentous biopolymer networks. The Journal of Cell Biology.

[CR38] Jensen OE, Revell CK (2023). Couple stresses and discrete potentials in the vertex model of cellular monolayers. Biomechanics and Modeling in Mechanobiology.

[CR39] Khunsaraki GM, Oscuii HN, Voloshin A (2021). Study of the mechanical behavior of subcellular organelles using a 3d finite element model of the tensegrity structure. Applied Sciences (Switzerland).

[CR40] Kojima H, Ishijima A, Yanagida T (1994). Direct measurement of stiffness of single actin filaments with and without tropomyosin by in vitro nanomanipulation. Proceedings of the National Academy of Sciences.

[CR41] Kovac B, Teo JL, Mäkelä TP (2013). Assembly of non-contractile dorsal stress fibers requires $$\alpha$$-actinin-1 and rac1 in migrating and spreading cells. Journal of Cell Science.

[CR42] Kreplak L, Fudge D (2007). Biomechanical properties of intermediate filaments: From tissues to single filaments and back. BioEssays.

[CR43] Krishnan R, Park CY, Lin YC (2009). Reinforcement versus fluidization in cytoskeletal mechanoresponsiveness. PLoS ONE.

[CR44] Latorre E, Kale S, Casares L (2018). Active superelasticity in three-dimensional epithelia of controlled shape. Nature.

[CR45] Lee SH (2020) Stroke Revisited: Pathophysiology of Stroke, 1st edn. Springer, Singapore, 10.1007/978-981-10-1430-7

[CR46] Lekka M, Gnanachandran K, Kubiak A (2021). Traction force microscopy - measuring the forces exerted by cells. Micron.

[CR47] Lieleg O, Claessens MMAE, Bausch AR (2010). Structure and dynamics of cross-linked actin networks. Soft Matter.

[CR48] Liu T, Guevara OE, Warburton RR (2010). Regulation of vimentin intermediate filaments in endothelial cells by hypoxia. Am J Physiol Cell Physiol.

[CR49] Lorenz C, Forsting J, Schepers AV (2019). Lateral subunit coupling determines intermediate filament mechanics. Physical Review Letters.

[CR50] Mann JM, Lam RHW, Weng S (2012). A silicone-based stretchable micropost array membrane for monitoring live-cell subcellular cytoskeletal response. Lab on a Chip.

[CR51] Martins JAC, Pato MPM, Pires EB (2006). A finite element model of skeletal muscles. Virtual and Physical Prototyping.

[CR52] Matthews BD, Overby DR, Alenghat FJ (2004). Mechanical properties of individual focal adhesions probed with a magnetic microneedle. Biochemical and Biophysical Research Communications.

[CR53] Mauri A, Hopf R, Ehret AE (2016). A discrete network model to represent the deformation behavior of human amnion. Journal of the Mechanical Behavior of Biomedical Materials.

[CR54] McEvoy E, Sneh T, Moeendarbary E (2022). Feedback between mechanosensitive signaling and active forces governs endothelial junction integrity. Nature Communications.

[CR55] McGrath JL, Osborn EA, Tardy YS (2000). Regulation of the actin cycle in vivo by actin filament severing. Proceedings of the National Academy of Sciences.

[CR56] Mosaffa P, Rodríguez-Ferran A, noz JJM, (2018) Hybrid cell-centred/vertex model for multicellular systems with equilibrium-preserving remodelling. International Journal for Numerical Methods in Biomedical Engineering 34:e2928. 10.1002/cnm.2928 10.1002/cnm.2928 28898926

[CR57] Nair RPH, Menon R, Kemkemer R (2021). Generalised image processing method for quantitative analysis of nucleus, cell and focal adhesion clusters. Current Directions in Biomedical Engineering.

[CR58] Nedelec F, Foethke D (2007). Collective langevin dynamics of flexible cytoskeletal fibers. New Journal of Physics.

[CR59] Nestor-Bergmann A, Johns E, Woolner S (2018). Mechanical characterization of disordered and anisotropic cellular monolayers. Physical Review E.

[CR60] Noll N, Mani M, Heemskerk I (2017). Active tension network model suggests an exotic mechanical state realized in epithelial tissues. Nature Physics.

[CR61] Øie CI, Mönkemöller V, Hübner W, et al (2018) New ways of looking at very small holes - using optical nanoscopy to visualize liver sinusoidal endothelial cell fenestrations. Nanophotonics p 575-596. 10.1515/nanoph-2017-0055

[CR62] Pensalfini M, Golde T, Trepat X (2023). Nonaffine mechanics of entangled networks inspired by intermediate filaments. Phys Rev Lett.

[CR63] Picu C, Ganghoffer JF (2020). Mechanics of Fibrous Materials and Applications, vol 596. Springer, Cham, Switzerland,.

[CR64] Picu RC (2011). Mechanics of random fiber networks - a review. Soft Matter.

[CR65] Popov K, Komianos J, Papoian GA (2016). MEDYAN: Mechanochemical simulations of contraction and polarity alignment in actomyosin networks. PLOS Computational Biology.

[CR66] Reyes Lúa MA (2020) Factors influencing the analysis of cell-substrate interaction, Diss. ETH No. 26609. PhD thesis, ETH Zurich, 10.3929/ethz-b-000440590

[CR67] Roth GA, Abate D, Abate KH (2018). Global, regional, and national age-sex-specific mortality for 282 causes of death in 195 countries and territories, 1980–2017: a systematic analysis for the Global Burden of Disease Study 2017. The Lancet.

[CR68] Ruiz-Franco J, Tauber J, van der Gucht J (2023). Cross-linker mobility governs fracture behavior of catch-bonded networks. Physical Review Letters.

[CR69] Schindelin J, Arganda-Carreras I, Frise E (2012). Fiji: An open-source platform for biological-image analysis. Nature Methods.

[CR70] Shen T, Shirinzadeh B, Zhong Y (2020). Sensing and modelling mechanical response in large deformation indentation of adherent cell using atomic force microscopy. Sensors (Switzerland).

[CR71] Silling SA (2000). Reformulation of elasticity theory for discontinuities and long-range forces. Journal of the Mechanics and Physics of Solids.

[CR72] Stracuzzi A, Dittmann J, Böl M (2021). Visco- and poroelastic contributions of the zona pellucida to the mechanical response of oocytes. Biomechanics and Modeling in Mechanobiology.

[CR73] Taylor M, Gözen I, Patel S (2016). Peridynamic modeling of ruptures in biomembranes. PLoS ONE.

[CR74] Thompson AP, Aktulga HM, Berger R (2022). LAMMPS - a flexible simulation tool for particle-based materials modeling at the atomic, meso, and continuum scales. Comp Phys Comm.

[CR75] Vajda J, Milojević M, Maver U (2021). Microvascular tissue engineering-a review. Biomedicines.

[CR76] Vignaud T, Copos C, Leterrier C (2021). Stress fibres are embedded in a contractile cortical network. Nature Materials.

[CR77] Wang M, Cai W, Yang AJ (2022). Gastric cancer cell-derived extracellular vesicles disrupt endothelial integrity and promote metastasis. Cancer Letters.

[CR78] Wu H, Shen Y, Sivagurunathan S (2022). Vimentin intermediate filaments and filamentous actin form unexpected interpenetrating networks that redefine the cell cortex. Proceedings of the National Academy of Sciences.

[CR79] Xu J, Xu X, Li X (2022). Cellular mechanics of wound formation in single cell layer under cyclic stretching. Biophysical Journal.

[CR80] Yan W, Ansari S, Lamson A, et al (2022) Toward the cellular-scale simulation of motor-driven cytoskeletal assemblies. eLife 11:e74160. 10.7554/eLife.74160 10.7554/eLife.74160 PMC9135453 35617115

[CR81] Yuan H, Huang C, Li J (2010). One-particle-thick, solvent-free, coarse-grained model for biological and biomimetic fluid membranes. Physical Review E - Statistical, Nonlinear, and Soft Matter Physics.

[CR82] Zancla A, Mozetic P, Orsini M (2022). A primer to traction force microscopy. Journal of Biological Chemistry.

[CR83] Zeng Y, Yip AK, Teo SK (2012). A three-dimensional random network model of the cytoskeleton and its role in mechanotransduction and nucleus deformation. Biomechanics and Modeling in Mechanobiology.

[CR84] Zhang J, Guan S (2020). Tensile properties of microtubules: A study by nonlinear molecular structural mechanics modelling. Physics Letters, Section A: General, Atomic and Solid State Physics.

[CR85] Zündel M, Ehret AE, Mazza E (2017a) Factors influencing the determination of cell traction forces. PLoS ONE 12:e0172927. 10.1371/journal.pone.0172927 10.1371/journal.pone.0172927 PMC5325618 28235004

[CR86] Zündel M, Mazza E, Ehret AE (2017). A 2.5d approach to the mechanics of electrospun fibre mats. Soft Matter.

