# Supplementary material for: Discrete network models of endothelial cells and their interactions with the substrate
Source: Biomech Model Mechanobiol. 2024 Feb 14;23(3):941–57. doi: 10.1007/s10237-023-01815-1 (PMC11101350; doi:10.1007/s10237-023-01815-1)
Supplement: Supplementary file 1 — (pdf 5893 KB) [file 10237_2023_1815_MOESM1_ESM.pdf]

Supplementary Material:

Discrete network models of endothelial cells and their interactions with the substrate

Raphael Jakob[1], Ben R. Britt[1,2], Costanza Giampietro[1,2], Edoardo Mazza[1,2], Alexander E. Ehret[1,2*]

[1]Institute for Mechanical Systems, ETH Zurich, CH-8092 Zürich, Switzerland.
[2]Empa, Swiss Federal Laboratories for Materials Science and Technology, CH-8600 Dübendorf, Switzerland.

*Corresponding author(s). E-mail(s): alexander.ehret@empa.ch;

A FE mesh refinement analysis

The dependence on the resolution of the FE model was analysed by refining the mesh. The results obtained with the mesh finally used for the calculations and with a mesh having finer discretisation along the z-direction and roughly double the number of elements showed little difference at a much higher computational cost (approx. 5-times). Fig. S1 shows the distribution of the difference $\Delta f^{(k)} = |\boldsymbol{f}^{(k)}| - |\boldsymbol{f}^{(k)}_{\text{refined}}|$ between the magnitudes of the forces computed with the original mesh and those obtained with the refined one for all FAs. A statistical analysis reveals that for 97% of the FAs the difference is below 5% of the mean force (1.8 nN) among the FAs, i.e. $-0.09\,\text{nN} \leq \Delta f \leq 0.09\,\text{nN}$. Given the drastic increase in computational time associated with a further decrease of the element size, these mesh-related errors were deemed acceptable.

B Equilibration of cell and substrate forces at FAs

To determine the displacements $\boldsymbol{u}^{(k)}$ of the FAs, the residuals $|\boldsymbol{R}^{(k)}|^2 = |\boldsymbol{f}^{(k)}_{\text{S}} + \boldsymbol{f}^{(k)}_{\text{C}}|^2$ formed by the reaction forces in the substrate model $\boldsymbol{f}^{(k)}_{S}$ and in the cell model $\boldsymbol{f}^{(k)}_{C}$ at each FA were minimised by a numerical procedure. The procedure was based on simplifying a Levenberg-Marquardt type algorithm (Chong and Žak, 2008, Ch. 9), which is described as follows.

B.1 Linearisation

Generally, the force $\boldsymbol{f}^{(k)}$ on a single FA k in both models depends on the displacements of all n FAs of the cell so that $\boldsymbol{f}^{(k)} = \boldsymbol{f}^{(k)}(\boldsymbol{u}^{(1)}, \boldsymbol{u}^{(2)}, \ldots, \boldsymbol{u}^{(n)})$. Taylor expansion about a set of given $\boldsymbol{u}^{(j)}_i$ for incremental changes of the FA displacements by $\Delta\boldsymbol{u}^{(j)}$ yields

$$\boldsymbol{f}^{(k)}(\boldsymbol{u}^{(1)}, ..., \boldsymbol{u}^{(n)}) = \boldsymbol{f}^{(k)}(\boldsymbol{u}^{(1)}_i, ..., \boldsymbol{u}^{(n)}_i) + \sum_{j=1}^{n} \left. \frac{\partial \boldsymbol{f}^{(k)}}{\partial \boldsymbol{u}^{(j)}} \right|_i \Delta\boldsymbol{u}^{(j)} + \mathcal{O}(\Delta\boldsymbol{u}^2), \tag{1}$$

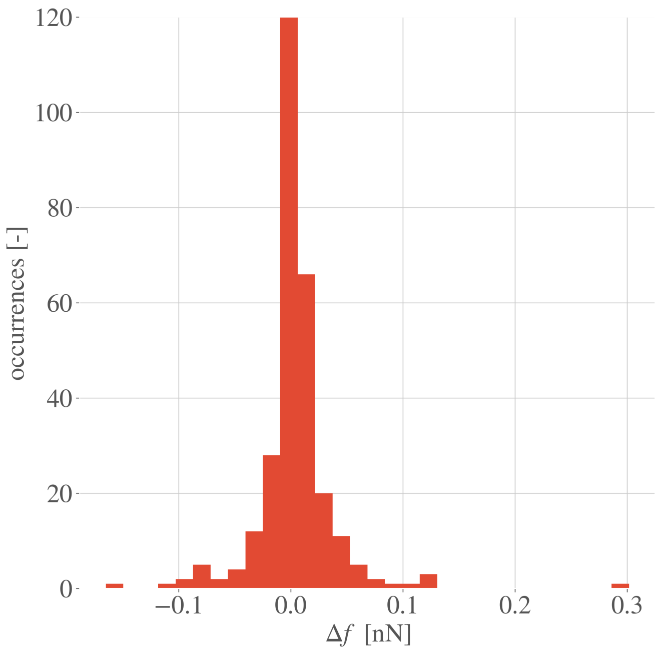

Fig. S1 Histogram of differences of computed forces at FAs $\Delta f^{(k)}$ between the present mesh and a refined one for all FAs

where $\Delta \boldsymbol{u}^{(j)} = \boldsymbol{u}^{(j)} - \boldsymbol{u}_i^{(j)}$. To simplify the procedure it was considered that the sensitivity of the force at FA k on the displacement of FA k itself is much larger than that of the displacements $\boldsymbol{u}^{(j \neq k)}$ at the other FAs. Therefore, the corresponding partial derivatives for $j \neq k$ were neglected, in addition to the higher order terms. The residual force vector $\boldsymbol{R}^{(k)}$ associated with a change $\Delta \boldsymbol{u}^{(k)}$ at each focal adhesion site was thus approximated as

$$\boldsymbol{R}^{(k)} \approx \underbrace{\left[\boldsymbol{f}_{\mathrm{C}}^{(k)}(\boldsymbol{u}_i^{(k)}) + \boldsymbol{f}_{\mathrm{S}}^{(k)}(\boldsymbol{u}_i^{(k)}) \right]}_{\boldsymbol{R}_i^{(k)}} + \underbrace{\left[\frac{\partial \boldsymbol{f}_{\mathrm{C}}^{(k)}}{\partial \boldsymbol{u}^{(k)}} + \frac{\partial \boldsymbol{f}_{\mathrm{S}}^{(k)}}{\partial \boldsymbol{u}^{(k)}} \right]_{\boldsymbol{u}_i^{(k)}}}_{\mathbf{J}_i^{(k)}} \Delta \boldsymbol{u}^{(k)}. \tag{2}$$

B.2 Update rule

Minimising the residual $|\boldsymbol{R}^{(k)}|^2$ and considering an iterative procedure with $\Delta \boldsymbol{u}_{i+1}^{(k)} = \boldsymbol{u}_{i+1}^{(k)} - \boldsymbol{u}_i^{(k)}$, leads to the well-known update rule of the Gauss-Newton method (Chong and Žak, 2008, Ch. 9)

$$\Delta \boldsymbol{u}_{i+1}^{(k)} = -\left[\mathbf{J}_i^{(k)}\right]^{-1} \boldsymbol{R}_i^{(k)} = -\left(\left[\mathbf{J}_i^{(k)}\right]^{\mathrm{T}} \left[\mathbf{J}_i^{(k)}\right] \right)^{-1} \left[\mathbf{J}_i^{(k)}\right]^{\mathrm{T}} \boldsymbol{R}_i^{(k)}. \tag{3}$$

To stabilise the iterations in case of (close-to) singular Jacobians $\mathbf{J}^{(k)}$, this rule was amended towards a Levenberg-Marquardt type update rule as (Chong and Žak, 2008, Ch. 9)

$$\Delta \boldsymbol{u}_{i+1}^{(k)} = -\left(\left[\mathbf{J}_i^{(k)}\right]^{\mathrm{T}} \left[\mathbf{J}_i^{(k)}\right] + \mu_i \mathbf{I} \right)^{-1} \left[\mathbf{J}_i^{(k)}\right]^{\mathrm{T}} \boldsymbol{R}_i^{(k)}, \tag{4}$$

where $\mathbf{I}$ is the identity matrix and μ_i is a parameter, which was initialised to $\mu_0 = $ 1e-3, and updated for $i \geq 1$ according to

$$\begin{aligned} \mu_{i+1} &= \mu_i / 2 \qquad \text{if } R_i^{(k)} \leq R_{i-1}^{(k)} \\ \mu_{i+1} &= 2\,\mu_i \qquad \text{if } R_i^{(k)} > R_{i-1}^{(k)}, \end{aligned} \tag{5}$$

where $R_i^{(k)} = |\boldsymbol{R}_i^{(k)}|$.

B.3 Initialisation and approximate computation of the Jacobian

The initial guess $\boldsymbol{u}_1^{(k)}$ was obtained by a preliminary DNM simulation in LAMMPS, assuming that the substrate is not deformed by the cell, leading to a preliminary guess $\boldsymbol{u}_0^{(k)}$ for all k. The resultant forces $\boldsymbol{f}_{\mathrm{C},0}^{(k)}$ at the FAs due to cytoskeletal activation were then imposed as concentrated loads $\boldsymbol{f}_{\mathrm{S},1}^{(k)}$ on the reference points in the substrate model. The FE analysis then provided the initial guess $\boldsymbol{u}_1^{(k)}$ to start the iterative scheme (Eq. 4). The partial derivatives in Eq. (2) were estimated by finite difference schemes in each step i, perturbing either the x or y-component of the displacement vector $\boldsymbol{u}_i^{(k)}$, and computing the corresponding changes in both components of the force vectors $\boldsymbol{f}^k$. For the DNM, this required $2n$ additional, but very fast simulations to compute the changes in $\boldsymbol{f}_{\mathrm{C}}^k$. For the FE model, it was again assumed that the FAs interact only weakly, so that only 2 simulations were performed, perturbing either all x or all y-components of the displacement vectors $\boldsymbol{u}^{(k)}$ for all $k = 1, 2, ..., n$ at once.

B.4 Reduction of the FA specific residuals

Fig. S2 shows the distribution of the single cell residuals $R_i^{(k)}$ at the 1st ($i = 1$), 3rd ($i = 3$) and 10th ($i = 10$) iteration for both the 0% (a) and 10% (b) substrate area strain case. The distributions reveal that the vast majority of residuals fall in the fist bin ($\leq 0.1\,\mathrm{nN}$), and a closer analysis revealed that 60% of the remaining residual results from less than 5% of the FAs with higher error remaining. This behaviour was similar for all $n = 5$ cells investigated, and $\bar{R}^{\mathrm{cell}}$ was reduced to 7.4% of its initial values for the unstretched and 5.5% for the stretched substrates within the 10 steps.

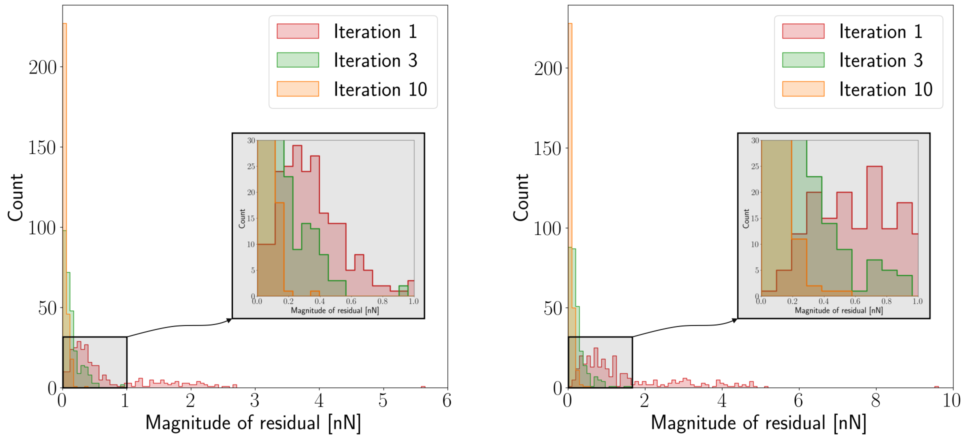

(a) (b)

Fig. S2 Reduction of residual with iterations. Distribution of the residuals $R^{(k)}$ at the $k = 1, ..., n$ FAs at iteration $i = 1, 3, 10$ for both substrate strains of 0% (a) and 10% (b)

C Traction force microscopy on HFFs

As experimental data also existed for human foreskin fibroblasts (HFFs), the model for the EC, that is described in Sec. 2.2.1 of the main part, was slightly modified towards representing features of HFFs. Fig. S3 shows the actin cytoskeleton of three adherent HFFs as well as their nuclei and illustrates that HFFs typically feature a larger number of stress fibers than ECs. Inspired by this, the number of ventral stress fibers was increased from 35-40 (as for the HUVEC representation) to 105-110 in the DNM. One of the thus achieved models is shown in Fig. S4a and the minimised energy configuration in S4b. Subsequent coupling of the HFF DNM and FE analysis and post-processing yielded area strains as shown in Fig. S5. The area strain difference

for each cell is listed in Tab. S1 and the histograms for one cell as well as the comparison of the simulation results with the experiments are shown in Fig. S6. Finally, the cell-scale residual $\bar{R}^{\text{cell}}$ resulting from the iterative minimisation scheme (Suppl. Mat. A and Sec. 2.5 of the main part) is documented in Tab. S2.

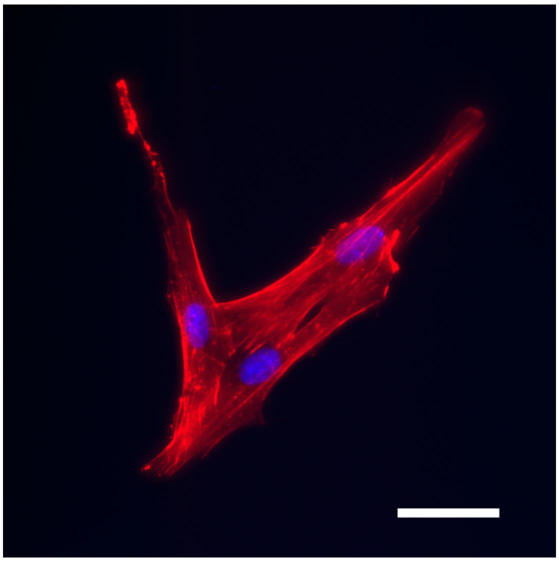

Fig. S3 Confocal laser scanning microscopy image of HFFs. 3 adherent cells with actin (red) and nuclei (DAPI, blue). The scale bar corresponds to 25 μm

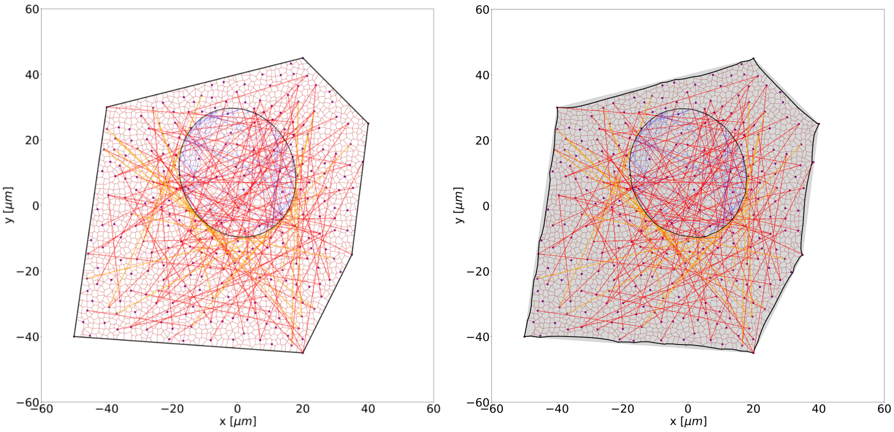

(a) $n_{\text{FA}} = 273$, $n_{\text{SF}} = 105$

(b) LAMMPS result

Fig. S4 DNMs of HFFs. (a) Reference configuration and (b) minimal energy configuration of DNM. The grey area denotes the domain occupied by the DNM in reference configuration. n_{FA} is the number of FAs and n_{SF} the number of SFs

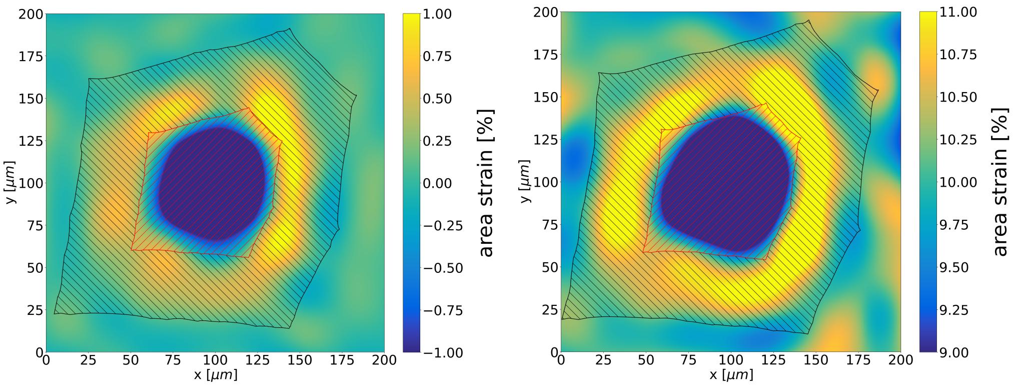

(a) (b)

Fig. S5 In-silico replication of TFM method by Reyes Lúa (2020): (a) Local area strain after DIC with bUnwarpJ for 0% and (b) 10% overall substrate strain. Hatching in red and black indicates underneath-cell and cell-vicinity areas, respectively

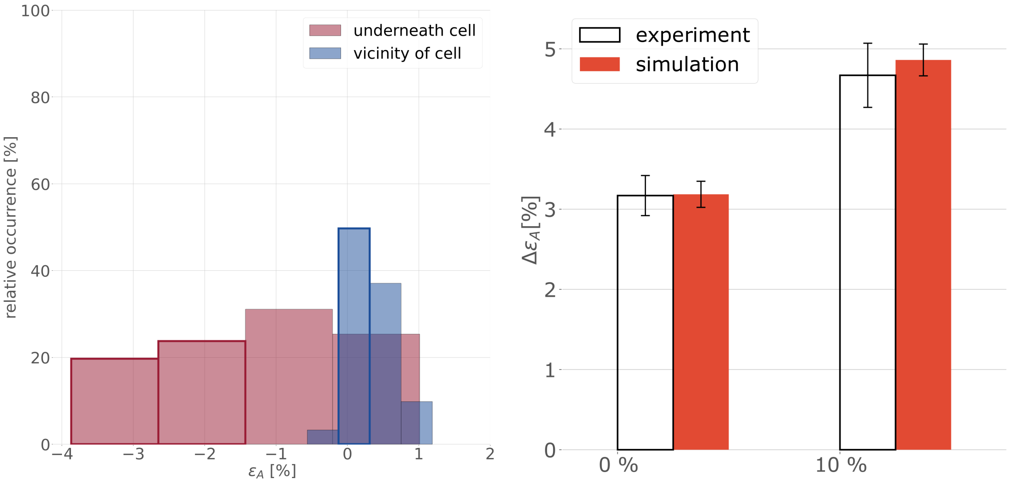

(a) (b)

Fig. S6 Evaluation of the area strain difference $\Delta\varepsilon_A$ for HFFs. (a) Normalised histograms of binned pixel-wise area strain obtained from the hatched regions in Fig. S5a for 0% global area strain. Red bins indicate underneath-cell strain, blue bins the area strain in the cell vicinity. Framed bins were used for the evaluation described in Sec. 2.6.1 of the main part. (b) comparison of the calculated area strain difference from the simulation with the ones reported by Reyes Lúa (2020) for the two load cases

	0% [%]	10% [%]
Cell 1	3.36	5.25
Cell 2	2.69	4.56
Cell 3	3.52	5.01
Cell 4	3.29	5.33
Cell 5	2.84	4.16
mean	3.14	4.86

Table S1 Results for area strain difference for five HFFs for the two loadcases

Subst. Area Strain	0%	10%
Cell 1	2.77	5.69
Cell 2	2.57	3.91
Cell 3	2.63	4.31
Cell 4	3.23	5.09
Cell 5	2.63	4.02
mean	2.77	4.60

Table S2 $\bar{R}^{\text{cell}}$ as described in section 2.5 of the main part in units of pN for each HFF model under 0% and 10% substrate area strain

D Influence of artificial fluorescent markers for strain analysis

Due to the only 8×8 intervals of the B-spline grid used to generate the locally varying deformation field of the substrate, the influence of the choice of artificial 'markers' was analysed. To this end, different sets of the superficial finite element mesh nodes were used for the generation of the fluorescent markers, grey scale images were produced (cf. Fig. 9a), the analysis of $\Delta\varepsilon_\mathrm{A}$ was repeated, and the effect was quantified.

More precisely, to achieve random selections of the markers from the substrate surface mesh nodes, we used different seed numbers in Python's random generators. The newly generated grey scale images were then analysed as described in the main part. This process was repeated twice to yield a total of three results for the area strain difference for the 0% and the 10% substrate strain case. The comparison of the three results to the experimental data is displayed in Fig. S7. The observed variations are smaller or at least in a similar magnitude as the variations observed upon a change of the model parameters in Fig. 11.

Consequently, the choice of the random set of markers is not expected to significantly affect the outcome of our study in terms of the 'overall' measure $\Delta\varepsilon_\mathrm{A}$. However, we critically remark that the B-spline interpolations of the area strain field itself may differ more strongly when using different grid points.

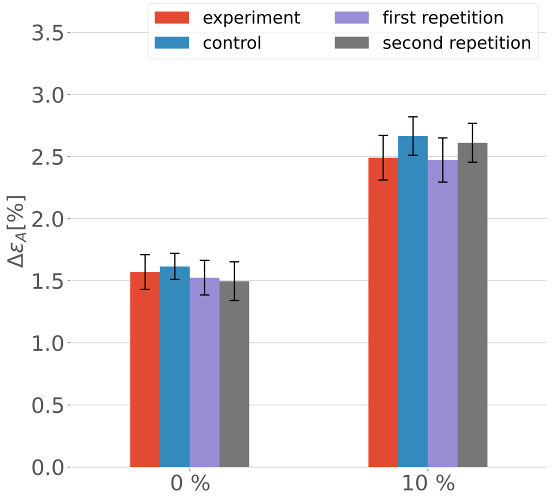

Fig. S7 Comparison of the results achieved with different selections of the random set of markers used for strain interpolation. The control case is analysed in the main part

References

Chong EKP, Žak SH (2008) An Introduction to Optimization. John Wiley & Sons, Ltd, Hoboken, NJ, https://doi.org/10.1002/9781118033340

Reyes Lúa MA (2020) Factors influencing the analysis of cell-substrate interaction, Diss. ETH No. 26609. PhD thesis, ETH Zurich, https://doi.org/10.3929/ethz-b-000440590
